# Organizational and psychological features of successful democratic enterprises: A systematic review of qualitative research

**DOI:** 10.3389/fpsyg.2022.947559

**Published:** 2022-11-04

**Authors:** Christine Unterrainer, Wolfgang G. Weber, Thomas Höge, Severin Hornung

**Affiliations:** Institute of Psychology, Applied Psychology – Work and Organizational Psychology, University of Innsbruck, Innsbruck, Austria

**Keywords:** democratic enterprises, organizational democracy, workplace democracy, worker cooperatives, retention, degeneration, regeneration

## Abstract

In organizational psychology the positive effects of democratically structured enterprises on their employees are well documented. However, the longstanding viability as well as economic success of democratic enterprises in a capitalistic market environment has long been contested. For instance, this has given rise to widespread endorsement of the “degeneration thesis” and the so-called “iron law of oligarchy”. By reviewing 77 qualitative studies that examined 83 democratic enterprises (including 15 studies on nine enterprises of the Mondragon Cooperative Cooperation network) within the last 50 years, the present systematic review provides evidence that such enterprises are able to economically survive and prosper. The majority of studied enterprises (63.5%) either resisted pressures toward degeneration or subsequently regenerated after undergoing degenerative processes. Only 9.5% fully degenerated in accordance with the degeneration thesis and the “iron law of oligarchy”, while 27.0% of the democratic enterprises showed diverse and mixed forms of degeneration tendencies, indicating that the notion of an “iron law” needs to be revised. Within the nine investigated cases of Mondragon not one single enterprise or group fully degenerated. Three cases showed degenerative tendencies, another three one degeneration tendencies and simultaneously regeneration, one case fully resisted degeneration tendencies (retention) and two cases regenerated. Further, this systematic review provides an overview of organizational and external conditions, non-/democratic or non-/participative practices and psychological phenomena that contribute to the degeneration, regeneration, or resistance to degeneration (i.e., retention). The described examples of such practices may help practitioners to implement and maintain democratic structures and processes in contemporary organizations.

## Introduction

*Democratic enterprises* are characterized by binding organizational structures and processes that entitle a substantial number of employees (at least one third) to participate directly (e.g., in general assemblies, meetings, or votes) or through elected representatives (e.g., on representative boards) in decision-making on strategic and tactical issues (Wegge et al., 2010; Unterrainer et al., 2011; Weber et al., 2020). Worker cooperatives, employee stock ownership plans (ESOPs, if owned by the majority of workers), democratic reform enterprises practicing representative participation and those social enterprises that employ core ideas of deliberative democracy, are prime examples of democratic enterprises. Recently, a comprehensive meta-analysis of quantitative studies has confirmed positive relationships between different types of democratic structures and participatory practices in organizations and positive employee outcomes, such as a perceived supportive climate, civic and prosocial (work) orientations, organizational commitment, and job involvement or work motivation (Weber et al., 2020).

Positive effects of democratic enterprises on employees and society as a whole are more or less well recognized among organizational scholars (see Parker, 2017; Weber et al., 2020). However, the economic success and viability of democratic enterprises, especially of worker cooperatives are more controversial.

### Degeneration thesis

Several sociologists and economists have voiced pessimistic views, questioning the potential for economic success and contesting the practical viability of democratic enterprises in a capitalist market environment (see overviews by Cornforth, 1995; Bretos et al., 2020). Specifically, the *degeneration thesis* by Webb and Webb (1914) posits that:

“[W]orker cooperatives will have to adopt the same organizational forms and priorities as capitalist businesses in order to survive. As a result, it is argued that cooperatives will gradually become dominated by a managerial elite who will effectively take decisions in the cooperative and so undermine democracy and the influence that other workers can exert” (Cornforth, 1995, p. 488).

On the one hand, the principles of capitalistic market economies will put pressure on democratic companies to adapt their internal structure accordingly (isomorphism). On the other hand, Webb and Webb (1914; according to Cornforth, 1995) stated that the worker-owners in worker cooperatives would demonstrate bad work discipline, a lack of operational knowledge and an unwillingness to adopt technical innovations, because they were masters who ruled their own enterprise. In the longer term, because of decreasing efficiency, democratic enterprises would fail economically or be forced to adopt capitalistic, non-democratic forms of ownership and management.

Strengthening Webbs' argumentation and focusing on internal organizational mechanisms of degeneration, Michels' (1915) “iron law of oligarchy” asserts that all democratic structures will eventually become dominated by powerful elites. This may happen because employees experience a need for leaders. Further, a growing size of a firm makes participative communication, the resolving of conflicts, and quick decisions in democratic meetings difficult, and favors the appointment of a permanent senior management in order to assure consistent corporate governance. Finally, technical specialization requires corresponding experts who gain strong organizational power because of their specific knowledge. Thus, an oligarchy of technological and management specialists will develop, who progressively evade democratic control, thus making their dominance and ruling permanent (Cornforth, 1995; Diefenbach, 2019).

Meister (1984) incorporated this proposition into his *cooperative's life cycle model*. Accordingly, the process of democratic degeneration comprises four stages: conquest, economic consolidation, coexistence, and administrative power (Bretos et al., 2020). The first stage, *conquest*, marks the starting point of a cooperative, characterized by direct democratic decision-making, high idealism, and commitment, as well as common economic, social and cultural goals shared by a small cohesive group of members. Economic functions are still poorly established and governing bodies not well defined. The second stage, *economic consolidation*, is one of transition, when conventional principles of organization develop and initial idealism decreases. Conflicts between egalitarian idealists and managers escalate, strengthening the power of management. In the third stage, *coexistence*, the cooperative loses its radical ideals and social goals, accepting market values instead. This phase is characterized by increases in size, division of labor, and role specialization. Direct democratic practices erode and representative boards are installed, leading to greater distance between managers and regular members. In the fourth stage, *administrative power*, members and representatives lose their influence, because economic logics dominate and an elite of managers takes full control (Meister, 1984; Cornforth, 1995; Bretos et al., 2020).

In his more recent conceptual overview, Diamantopoulos (2012) argues that worker cooperatives suffer from their dual nature, namely from a structural tension between their function as economic enterprise, on the one hand, and their identity which represents a democratic organization following social goals, on the other hand. The longer cooperatives operate economically, the more they grow, and the more the value-oriented, idealistic founders meet a younger generation of pragmatists, the more conflicts between economic and social goals will arise. Market pressures from economic rivals will increasingly favor the primacy of economic goals and the power of in-house experts to impose capitalist accounting and management methods. According to Diamantopoulos (2012), mature cooperatives are tempted to downplay their socio-political objectives to attract customers and sponsors. Both goal and organizational degeneration, namely, the removal of structures of democratic decision-making and collective ownership, as well as an erosion of the cooperative culture including its democratic values, is becoming increasingly likely. What is more, those large and economically successful cooperatives which undergo a degenerative process may transfer their decline in values to regional cooperative associations and, thus, contribute to a further spread of degeneration.

Additionally, Chaves and Sajardo (2004) analyze the role that managers play in the process of organizational degeneration. They refer to two premises that underlie Michels' (1915) and, following him, Meister's theories: First, social and professional leadership abilities are unequally distributed among the members of a democratic organization. Thus, only a minority of the members possesses strong managerial capabilities. Second, with increasing size and complexity of democratic organizations operating within a competitive market, there is growing pressure for permanent management positions to be created. Persons with comprehensive technological and organizational knowledge will occupy permanent management positions, and will progressively gain control of strategic information, intangible strategic company assets (e.g., exploiting social networks), and experience-based leadership expertise. If those management experts were educated in the spirit of neoliberal ideology, then they will counteract and transform the social and democratic culture. Their growth of expert power will supersede the participation of other employee-owners or their representatives in strategic organizational decision-making. This happens because the latter lack sufficient professional knowledge and social/communicative competence to act as a counterweight. Rather, employee representatives in government councils of democratic enterprises are captured by managers' influence tactics inducing co-opting and connivance (Chaves and Sajardo, 2004).

Following those authors' conceptual considerations, we will review the existing studies with respect to what proportion degeneration takes among the documented cases. Furthermore, we will investigate which of the described conditions, practices, and psychological phenomena that contribute to the degeneration of organizational democracy were observed empirically and whether others, not yet described, can be found, too. Accordingly, Research Question 1 reads:

RQ1: What organizational and external conditions, non-democratic or non-participative practices, and psychological phenomena contribute to the degeneration of democratic enterprises?

### Retention thesis

Criticizing the deterministic character of the degeneration thesis, Rothschild-Whitt (1976; cf. Rothschild-Whitt and Whitt, 1986) introduced the *retention* thesis (also called: maintenance thesis) in demonstrating that democratic modes of organizations are conditional. Rothschild-Whitt argued that under favorable conditions, democratic enterprises can retain structures and practices of direct democracy and maintain adherence to cooperative, democratic, and humanistic values. Retention does not imply that there are no changes in structural elements, principles, procedures, and participative practices of an organization. Rather, retention means that there are limited changes, which prevent processes of degeneration of the respective democratic enterprise.

Several scholars identified conditions and practices through which democratic enterprises resisted degenerative developments. This was done either theoretically in conceptual essays or empirically in (limited) narrative reviews (see Rothschild-Whitt, 1976; Cornforth, 1995; Bretos et al., 2020; and below).

*Organizational conditions* which were reported in these reviews included structural aspects, such as

- a small size of the company,
- alternative growth patterns (e.g., fission of the company),
- and appropriate technology that does not require a rigid division of labor (Rothschild-Whitt, 1976).

*Democratic and participative practices:* Frequently identified in previous reviews and conceptual essays were practices that aim to foster and maintain co-operative principles and ideals by sustaining the democratic values and culture. These include:

- “Cooperativization” of capitalist subsidiaries: The transformation of hierarchically structured enterprises purchased by a democratic parent enterprise into democratic companies may prevent a degenerative process of the respective democratic enterprise group (Bretos et al., 2020).
- Linking the social goals of the enterprise to wider social movements and coordinating the activities with neighboring communities can sustain an orientation of organizational members toward achieving a transition of the capitalist economic system into a more communitarian or alternative-socialist economy (Rothschild-Whitt, 1976; Pineiro Harnecker, 2009; Diamantopoulos, 2012; Bretos et al., 2020).
- Complementarily, having a base in professionals from the regional or local community, who support the democratic enterprise in providing their knowledge or political assistance, or buying its products, will counteract competitive pressure on the market (Rothschild-Whitt, 1976; Diamantopoulos, 2012).
- Further practices to maintain democratic values are institutionalized opportunities for open criticism and discussions among employees (including self-criticism), as well as between hierarchical levels in forums to prevent “oligarchization” (Rothschild-Whitt, 1976; Cornforth, 1995; Eikenberry, 2009).
- In line with a culture of open discussion, institutionalizing the re-enactment of democratic discourse focusing on issues of democracy, social transformation, and community development is considered a promising practice to internalize co-operative principles among employees (Eikenberry, 2009; Bretos et al., 2020).
- Value-based processes of common recruitment, personnel selection, and induction procedures, institutionalization of cooperative education and training, and centrality of a thorough socialization of members (especially also future managers) with regard to cooperative values are suggested to contribute to the cultivation of shared meaning and commitment to the co-operative's aims and practices (Cornforth, 1995; Chaves and Sajardo, 2004; Sauser, 2009; Bretos et al., 2020).
- Transparency, knowledge- and information sharing in addition to the aforementioned HRM methods, periodic rotation of staff among departments and jobs, and task sharing, are suggested as ways of spreading professional skills, expertise, and experience within the workplace as an internal resource base (Rothschild-Whitt, 1976; Cornforth, 1995; Bretos et al., 2020).
- A further principle to prevent degeneration concerns organizing of work in ways to maximize broad-based participation on the shop-floor (e.g., self-managed work groups) and at the management-level to foster a democratic and participatory culture grounded in self-determination, egalitarianism, and solidarity among workers (Hernandez, 2006; Sauser, 2009; Bretos et al., 2020).
- Additionally, democratic and supportive leadership on the level of the organizational units (e.g., departments and work groups) will provide psychological safety and opportunities for the organizational members to participate directly in operational or tactical decision-making and, thus, counteract degenerative tendencies (Sauser, 2009).
- Finally, integrating representatives of the concerned union into democratic decision-making and taking care for their role to protect the employees' interests can help to prevent an erosion of the democratic processes (Sauser, 2009).

Referring those authors' conceptual considerations and narrative reviews, we are interested in available studies with regard to what proportion retention takes among the documented cases. Furthermore, we will investigate which of the described conditions and practices that contribute to the retention of organizational democracy can be observed empirically and whether others, not yet described, can be identified. We will also consider associated psychological phenomena and formulate the following Research Question 2:

RQ2: What organizational and external conditions, democratic or participative practices, and psychological phenomena contribute to the retention of democratic structures and principles in an enterprise?

### Regeneration thesis

The dual nature of cooperatives means that they are both an economic enterprise and a democratic as well as a social association. Despite this inherent conflictual character, cooperatives have been shown to possess the potential to regenerate from degenerative processes (Diamantopoulos, 2012). For instance, Batstone's (1983) Life-Cycle Model of cooperatives is an alternative conception, assuming a resurgence or *regeneration* of organizational democracy. Accordingly, cooperatives initially start out with a system of direct (primitive) democracy. In the second phase, after the first crucial years, the financial basis will have developed and the co-operative is established. In this ensuing phase of *degeneration*, the original spirit and culture will be diluted over time, as the organization becomes more routinized and some members will serve in specialized managerial functions. As the organization increases in size, new workers will be recruited not all of which will become full members of the cooperative. As a result, the extent of direct democracy declines and new forms of representative bodies and institutions are established. This is regarded as “the low point of democracy”. In the third and last stage, a *regeneration* (of democracy) can occur. This regeneration is often not a return to the initial form of direct democracy, but associated with a decline in the dominance of professional management and a re-focusing on the interests of workers, as opposed to an economic emphasis on capital or profits. After this final stage is completed, another cycle can commence (Batstone, 1983, p. 150ff; Bretos et al., 2020, p. 439).

The mechanisms regarding how democratic enterprises can regenerate from degenerative tendencies may be similar to the practices promoting retention of organizational democracy described before. In his narrative review, Diamantopoulos (2012, p. 211) emphasizes the crucial role of the reconstruction of mobilizing social economy networks, multi-stakeholder cooperatives acting as institutional intermediaries, visionary social movement entrepreneurs providing value-activating leadership, and educational and cultural interventions (e.g., fostering engagement for the local economy, creating inter-cooperative solidarity) in the regeneration process. Batstone (1983) pointed out the importance of trainings with regard to co-operative values and Cornforth (1995) and Bretos et al. (2020) refer to the importance of revitalizing the democratic culture as a central practice of regeneration or retention. Revitalizing or preserving the democratic culture and social objectives requires the creation of professional associations and training institutions. Those provide professional education and help to teach comprehensive knowledge and skills concerning democratic, social, and cooperative values and purposes of social economy enterprises for managers. These support organizations should also help democratic companies to recruit or internally select suitable managers (see a conceptual overview by Chaves and Sajardo, 2004).

However, Bretos et al. (2020) criticize that proponents of the regeneration thesis assume a consistent and homogeneous process. More recent studies would emphasize the inherent paradoxical characteristics of cooperatives as members permanently struggle to find a balance between competing market and social demands (Ng and Ng, 2009; Storey et al., 2014; Narvaiza et al., 2017). This implies that cooperatives might undergo processes of *partial regeneration*, reverting only to some democratic organizational structures and practices, while others remain unaltered. In this vein, the cooperative can benefit from participatory and democratic processes, but continue to also have access to resources based on a market logic (Bretos et al., 2020, p. 440). Thus, regeneration may not be a consistent homogeneous process. On the contrary, degeneration and regeneration are not mutually exclusive, but can occur simultaneously.

In developing this theorizing on paradoxical tensions and partial regeneration Bretos et al. (2020) draw on and elaborate the previous life-cycle models by Batstone (1983) and Meister (1984). The revised process-oriented differentiation proposed by Bretos et al. (2020) is the most comprehensive and best developed model and particularly valuable for more in-depth empirical analyses of transformation processes in democratic organizations. Bretos et al.'s (2020) life-cycle model mirrors Meister's (1984) model in Stages 1 (conquest) to 4 (administrative power). The new Stage 5 proposes processes of regeneration—according to Batstone's (1983) model—but additionally includes pathways of institutional isomorphism (capitalistic solidification) or dissolution and exit from the industry. After regeneration, a new life-cycle may start and these processes seem to suggest more or less permanent changes and continuous passages from one stage to another. However, after institutional isomorphism and dissolution and exit the industry, the life cycle of a cooperative will end (Bretos et al., 2020).

According to the above-mentioned conceptual considerations of different scholars, we will review existing studies with respect to what proportion regeneration takes among the documented cases. Furthermore, we will investigate which of the described conditions and practices that contribute to the regeneration of organizational democracy can be observed empirically and whether others, not yet described, can be identified. We will also consider related psychological phenomena. Accordingly, Research Question 3 asks:

RQ3: What organizational and external conditions, democratic or participative practices, and psychological phenomena contribute to the regeneration of an enterprise?

### The Mondragon Cooperative Corporation—A special case

The Mondragon Cooperative Corporation is the biggest democratic network of worker cooperatives worldwide (see results - research question 4). Because of its vast size and complex organizational structure, enterprises belonging to this conglomerate will be treated in a separate research question:

RQ4: What forms of transformation and related organizational and external conditions, democratic or participative practices, and psychological phenomena can be found in the Mondragon Cooperative Cooperation network?

### Objectives and contributions of this review

To conclude, while a considerable number of studies on transformation processes of democratic enterprises have been conducted, no systematic review exists which indicates the proportion of democratic enterprises undergoing degeneration, regeneration, or retention. Available conceptual essays or narrative reviews each refer only to a rather limited (Sauser, 2009; Diamantopoulos, 2012; Lee and Edmondson, 2017; Diefenbach, 2019) or a corporation-specific (Bretos et al., 2020) selection of existing studies compared to the studies included within our review presented in the following. None of them applied systematic review methodology (e.g., providing criteria for minimal methodological standards of the included studies). We consider this research gap not only of great theoretical but also practical importance. Particularly, as, in additional to a secondary analysis and evaluation of proposed democratic transformation theses, we also seek to explore what external conditions, organizational practices and related psychological resources are associated with the identified processes of degeneration, regeneration, and resisting the degeneration (i.e., retention). Therefore, our aim is to close this research gap by providing a systematic review on relevant qualitative research studies (published between 1970 and 2020). We focus on qualitative studies, as these are most adequate to answer our research questions. The latter require the analysis of concrete transformation processes of organizational democracy and their accompanying participative or non-participative practices and psychological phenomena (representing resources or obstacles for a vital organizational democracy) in the specific context of the respective enterprises.

## Methods

### Identification of studies

**Figure 1:**
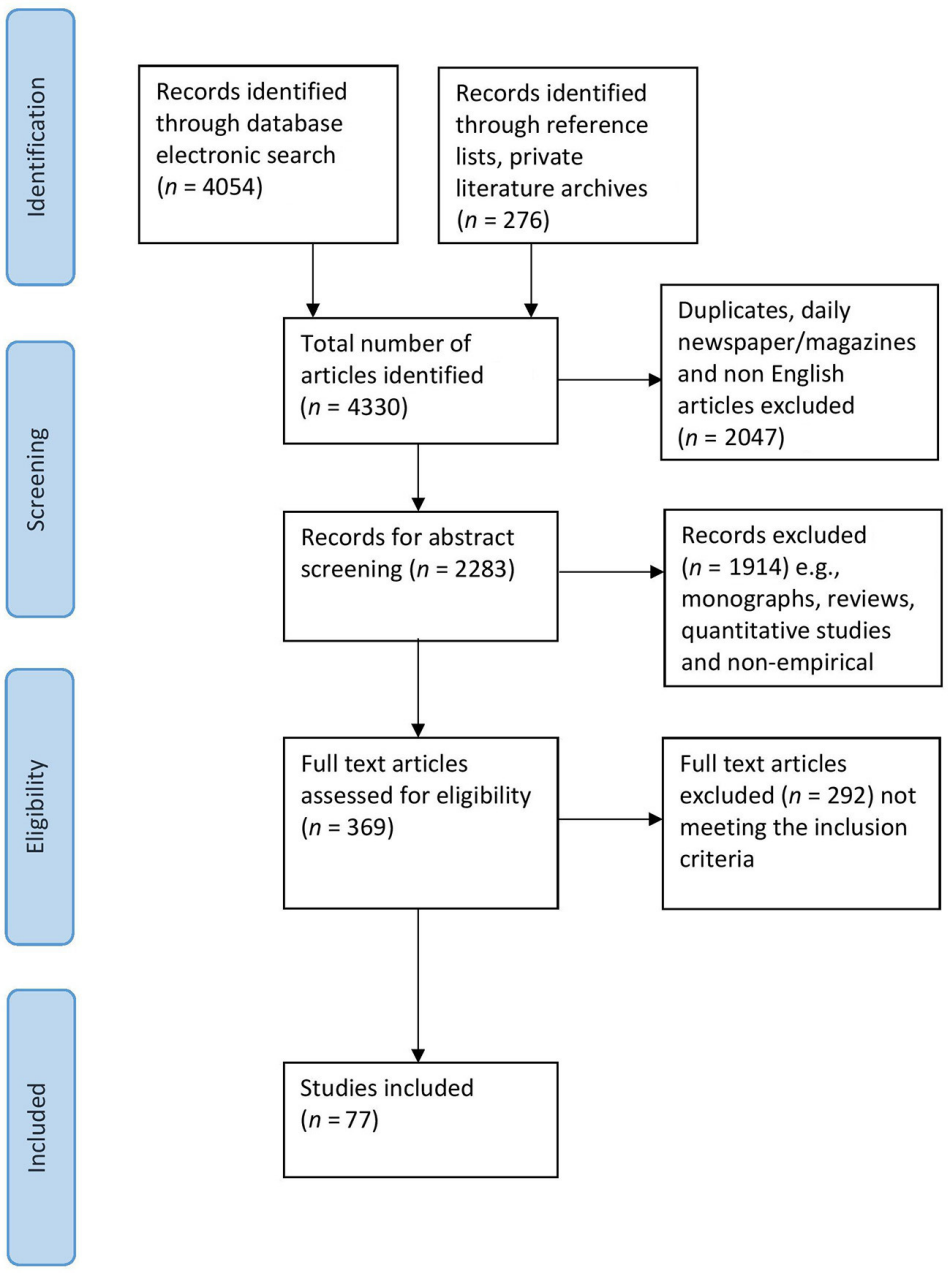
PRISMA flow diagram.

For conducting a systematic literature review on qualitative research studies examining democratic enterprises, the PRISMA model was used (Moher et al., 2009). The PRISMA flow diagram is depicted in Figure 1. Focusing on studies published between 1970 and 2020, the databases PsycINFO, PSYNDEX, PsycArticles, Business Source Premier, ECONLIT, ERIC, SOCINDEX, and Medline were searched, using 51 different search strings (e.g., “organizational democracy,” “industrial democracy,” “workers' self-management,” “worker co-operative,” “democratic firm”; the full list of search terms is provided in Supplementary Table 1). The starting point of 1970 was chosen, because a first wave of empirical studies on employee ownership emerged in the 1970ies (e.g., Obradovic, 1970; Kavcic et al., 1971; Goldstein, 1978; Long, 1978; Nightingale, 1979; Russell et al., 1979). Based on searches of the electronic database, initially 4,054 records were identified. Additionally, by scanning our own extensive literature archive and reference lists of all publications that met the inclusion criteria, 276 additional articles were included.

### Screening the literature

Out of the total number of identified articles (4,330), we excluded 2,047 duplicates, articles in daily newspaper or magazines and non-English language articles, thus, yielding 2,283 abstracts to be screened. Based on the abstracts, we further eliminated monographs, reviews, strictly quantitative studies, and non-empirical studies, resulting in 369 full text articles. We assessed full texts according to our inclusion and exclusion criteria, excluding another 292 articles that did not meet standards. Finally, 77 articles were included in the systematic review. These articles were published between 1980 and 2020 and investigated 74 enterprises, plus nine enterprise groups from the Mondragon Cooperative Corporation as a special case. Several identified studies published between 1970 and 1979 did not meet inclusion criteria.

### Inclusion and exclusion criteria

Inclusion and exclusion criteria were specified prior to the literature search. In terms of publication type, the focus was on qualitative journal or editorial book articles. Excluded, due to limited resources relative to the extensive number of publications, were monographs, unpublished dissertations, conference proceedings, gray literature, reviews, quantitative studies, non-empirical studies, and articles that were not accessible. Further excluded were articles that examined several democratic enterprises, but reported only general findings, such that no clear inference could be made with regard to the individual enterprises (e.g., Pineiro Harnecker, 2007, 2009, 2012; Brown et al., 2019). In the process of reviewing full-text articles, inclusion/exclusion criteria were further refined. Eventually, we included studies that met the following criteria:

a) Qualitative empirical studies that provide at least a minimum of information on methodology. This means, studies had to specify the number of conducted interviews and, in case of observational or ethnographic studies, the number of conducted observations and duration of the ethnographic field work.
b) To be included, publications needed to focus on issues of degeneration, regeneration, and/or retention of democratic structures and practices or investigate psychological and organizational concepts and features that enhance or impede retention, degeneration or regeneration of democratic enterprises.
c) Democratic structure and practices of the investigated enterprise(s) had to be described in sufficient detail. This means that studied organization(s) can be classified, at the time of their foundation, as a democratic enterprise according to Weber et al.'s (2020) classification criteria: the enterprise has a binding organizational decision-structure that entitles a substantial number of employees (at least one third) to participate directly (e.g., in general assemblies, meetings or votes) or through elected representatives (e.g., on representative boards) in making decisions on strategic or tactical issues (Unterrainer et al., 2011; Weber et al., 2020). Accordingly, networks of freelancers, traditional consumer co-operatives, as well as banking co-operatives and building cooperatives, in which <1/3 of employees possessed ownership and collective decision rights (or with missing data regarding worker ownership), were excluded.
d) A sample description of the studied enterprises has to be provided, specifying, at least, the country where the research was conducted, the year the organization was founded, the year when the democratic model was implemented, the number of employees, the distribution of ownership rights, and the examined branch(es) or division(s).
e) For a valid assessment of the retention thesis, studies are included only if the democratic model of the investigated enterprises has been in place for at least for 5 years. An exception to this criterion are studies reporting on enterprises which degenerated earlier than after 5 years of democratic functioning.

### Necessary refinement of different forms of degeneration, retention, and regeneration

According to the research question, the level of analysis is the individual enterprise. Thus, an enterprise represents one case, the empirical studies of which can encompass one or several publications. Based on Batstone's (1983) and Bretos et al.'s (2020) expanded Life-Cycle Model of cooperatives, we developed a retention-degeneration-regeneration classification scheme. Considering the sample of studies resulting from the systematic literature search, we elaborated this classification scheme by including “degeneration tendency” as an additional category. Thus, our primary classification comprised the four categories of “degeneration,” “degeneration tendency,” “retention,” and “regeneration”.

Further, we drew on Cornforth et al.'s (1988) work on three main degenerative dynamics in cooperatives to assess in which form (constitutional, organizational, or goal/democratic-cultural-values-driven) the respective enterprise degenerated or regenerated. The first form, “… *constitutional degeneration* involves cooperatives adopting capitalist forms of organization in which employees are excluded from the rights and benefits of cooperative membership” (Bretos et al., 2020, p. 438). This means that, while the number of employees increases over time, the number of working cooperative members or other employee-owners, entitled to organizational decision-making and participation in collective ownership, decreases. The second form, “… *organizational degeneration* implies that employee involvement in decision-making is diminished in favor of control by a managerial elite or technocracy.” (Bretos et al., 2020, p. 438). The third form, “… *goal/cultural degeneration*, entails the prevalence of the conventional business goals of profit seeking and growth over socially-oriented targets” (Bretos et al., 2020, p. 438, following Cornforth et al., 1988). This type of degeneration occurs, when democratic enterprises “are increasingly prioritizing profits or growth as their prime purpose” (Basterretxea et al., 2019, p. 587). Additionally, we also distinguished different degrees or specific combinations within these main categories, see our elaborated classification scheme in the following.

#### Degeneration

##### Constitutional degeneration

According to our conceptual elaboration, it is useful to distinguish three forms of *constitutional degeneration*. These forms are empirically interrelated but can be analytically separated:

- A *moderate constitutional degeneration tendency* is present when, although the proportion of employees without democratic rights has increased, the majority (50% or more) still possesses full ownership and collective decision rights.
- A *strong constitutional degeneration tendency* is attested when, after a decline in the proportion of employee-owners relative to the non-owning employees, only a minority of at least 1/3 of employees are still collective owners. This distinction was made because the exclusion of a majority of employees from participation in democratic decision-making represents a significant indication for a process of constitutional degeneration. The more employees are excluded, the smaller the proportion of staff who have opportunities for developing positive organizational attitudes and behaviors or community-related orientations through practicing democratic participation and responsibility (see reviews by Weber et al., 2020; Kim, 2021).
- A *full constitutional degeneration* applies when, after a decline of the proportion of employee-owners, only a minority of <1/3 of the employees participate in ownership of their enterprise (whereas at least 1/3 of the staff had been employee-owners before). In this case, a formerly democratic and employee-owned enterprise ends up very closely resembling an autocratically structured and managed firm, because a relatively small group of owners and managers exerts control.

##### Organizational degeneration

- A *organizational degeneration tendency* is defined as a change of the “gestalt” or form of democratic participation, specifically, when decisions that were formerly made in general employee assemblies (direct democracy), are delegated to councils, committees or boards (representative democracy).
- A *full organizational degeneration* is indicated by an increase in functional specialization, for example, in the form of experts and managers who are difficult to replace. Important strategic and tactical decisions, which used to be made in representative democratic bodies (or general assemblies), are referred to the decision-making power of a management elite, which is not elected (and cannot be voted out of office) by the employees. Although a non-binding say or consultative participation of employees may be sought, final decisions are determined by the management elite.

##### Goal/cultural degeneration

- A *form* of a *goal/cultural degeneration tendency* is characterized by increasing importance attributed to business objectives within organizational communication, planning, and decision-making, relative to social goals of a cooperative economy. Social goals comprise employment security, just or needs-related income, education and training in cooperative values, inter-organizational solidarity, retention of organizational democracy, quality of working life, creating socially useful products or services, or contributing to the local community (cf. Cheney, 1997; Flecha and Ngai, 2014; Bretos et al., 2020). This growing influence of economic relative to social goals in a democratic enterprise can be observed when the rhetoric of business economics is increasingly assimilated in the discourse among managers, among non-managing employees or between both groups (cf. Taylor, 1994; Cheney et al., 2004).
- A *full form* of a *goal/cultural degeneration* manifests in the observation that economic goals and values clearly dominate strategic decisions and shape intra-organizational communication at the expense of social objectives both among managers and between managers and non-managing employees.

#### Retention

In the case of *retention* of the democratic structure, we did not differ between constitutional, organizational and goal/cultural forms, based on a decision to apply a conservative analytical strategy. Thus, an enterprise was only included in the retention category if no indication for any form of degeneration or degeneration tendency was found.

Within the “retention” category, we differentiated between enterprises on which we only had *cross-sectional information* and enterprises for which we had *longitudinal information*. Several studies had investigated the same enterprise at different points in time. For example, a bicycle-hire and sales shop in Scotland was studied by Oliver (1984), Cornforth (1995), and Sacchetti and Tortia (2020). Such investigations provided us with longitudinal information on whether the enterprises' democratic structure and practices had remained stable or changed over time. Additionally, we included several studies that provided only cross-sectional information on the examined enterprise but did not report any organizational transformations that, possibly, had occurred in the past. For these studies, we were not able to describe the development, but only the status quo of democratic structures and practices at the time of investigation.

#### Regeneration

Our categorization of different forms of *regeneration* of organizational democracy follows Bretos et al.'s (2020) extension of Batstone's life-cycle model:

“A smaller but growing line of research has highlighted cooperatives' potential to *regenerate*; that is, to revive cooperative values and re-assemble democratically-structured forms of decision making as a reaction to degeneration” (e.g., Ng and Ng, 2009; Diamantopoulos, 2012; Giagnocavo et al., 2014; Storey et al., 2014; Narvaiza et al., 2017). “These studies illustrate how cooperatives can regenerate by mobilizing resources to trigger organizational change, such as by reinforcing a culture of discussion and open criticism and strengthening members' active participation.” (Bretos et al., 2020, p. 436)

Complementary to the three distinguished forms of degeneration (Cornforth et al., 1988), we also differentiate between three forms of regeneration.

##### Constitutional regeneration

*Constitutional regeneration* means that in a democratic enterprise that has gone through a process of constitutional degeneration, measures are taken to increase the proportion of employees who participate in democratic decision-making and collective ownership, such that eventually at least the majority of employees (50% or more) is able to participate in democratic processes and ownership.

##### Organizational regeneration

*Organizational regeneration* is attested, if in a democratic enterprise that has gone through a process of organizational degeneration, the dominance of managers or other experts in the democratic bodies of this firm is overcome *by changing at least some of the actors* participating in strategic and tactical planning and decision-making.

##### Goal/cultural regeneration

*Goal/cultural regeneration* applies when in a democratic enterprise that has gone through a process of goal/cultural degeneration, the dominance of a mindset of economistic values among management or employees has been overcome by facilitating a culture of humanistic, communitarian or cooperative goals and social values that are at least attributed a similar importance.

## Results

### Preliminary, descriptive results

Table 1 provides a summary of the classification of all *N* = 74 democratic enterprises investigated in the studies included in this systematic review (excluding the Mondragon Cooperative Corporation which is separately described in Section Research question 4: The Mondragon Cooperative Cooperation Network and its forms of transformation as well as relating conditions, practices, and psychological phenomena). This classification demonstrates how many of the studied democratic enterprises show indications for the degeneration thesis and the “iron law of oligarchy” and how many support the counterpropositions of retention and regeneration.

**Table 1:** Number of companies per transformation form (Mondragon Cooperative Corporation is not included).

**Transformation form**	**Number of enterprises**
**Full degeneration**	**(*****n*** **= 7)**
Full constitutional degeneration	2
Full organizational degeneration	0
Full goal/cultural degeneration	1
More than one form of full degeneration	4
**Degeneration tendency**	**(*****n*** **= 20)**
Moderate constitutional degeneration tendency (worker owner = majority)	7
Strong constitutional degeneration tendency (worker owner = minority)	1
Organizational degeneration tendency	5
Goal/cultural degeneration tendency	3
More than one form of degeneration tendency	4
**Retention**	**(*****n*** **= 39)**
Cross-sectional information	8
Longitudinal information	31
**Regeneration**	**(*****n*** **= 8)**
Constitutional regeneration	1
Organizational regeneration	3
Goal/Cultural regeneration	4

Overall number of enterprises N = 74.

Evidence for processes that amounted to *full degeneration* of democratic structures and processes was reported for *n* = 7 formerly democratic enterprises (9.5%). Out of those, *n* = 2 indicated full constitutional degeneration and *n* = 1 full goal/cultural degeneration. While no example of a full organizational degeneration was found by itself, *n* = 4 cases reported indications for more than one form of full degeneration. In one case, the Kompro (Kalmi, 2002) displayed full constitutional and full organizational degeneration, since the management team bought out all worker shareholders and the decision-making power was confined to this manager elite. The three other originally democratic enterprises underwent transformations into two investor-owned, and one family-owned firm, respectively.

Further, indications for *tendencies of democratic degeneration* were found in overall *n* = 20 cases (27.0%). These could further be subdivided into a moderate (*n* = 7) and strong (*n* = 1) constitutional degeneration tendency, an organizational degeneration tendency (*n* = 5) and a goal/cultural degeneration tendency (*n* = 3). Additionally, *n* = 4 democratic enterprises showed signs of more than one form of degeneration tendency. One experienced a slight organizational and goal/cultural degeneration tendency, another one a goal/cultural and moderate constitutional degeneration tendency, and the third one a goal/cultural and strong constitutional degeneration tendency.

By far the largest number of *n* = 39 democratic enterprises (52.7%) were allocated to the group that resisted any form of degeneration (classified as *retention*). Out of these, only cross-sectional information was available for *n* = 8 enterprises, whereas longitudinal information was available for *n* = 31 democratic enterprises.

Finally, a number of *n* = 8 cases of *regeneration* could be identified (10.8%), out of which *n* = 1 fulfilled the criteria for constitutional regeneration, *n* = 3 conformed with organizational regeneration, and *n* = 4 showed signs of goal/cultural regeneration.

Supplementary Tables 2–4 provide an overview of all identified enterprises between 1970 and 2020 (Mondragon Cooperative Corporation is not included) that were classified in terms of *full degeneration, degeneration tendency, retention*, and *regeneration*. The Tables also include information on the country where the respective site is located, the number of employees, the founding year of the company as well as the year of the introduction of the democratic model, the tenure of democratic practices, and the studies' authors. Additionally, Supplementary Tables 2–4 provide information about the proportion of employee-ownership in each enterprise. For the assessment of organizational democracy, it is relevant whether the majority of employees was participating in democratic governance at the time of study. Out of the 74 enterprises, in *n* = 62 enterprises (83.7%) the *majority of employees were collective owners*, in *n* = 4 enterprises (5.4%) ownership among workers was <50% (*minority were owners)*, but higher than 1/3, in *n* = 3 enterprises (4.1%) <1/3 of workers were owners; in two cases (2.7%) the description was not completely clear, but suggested that the majority of employees collectively owned the firm. However, *n* = 3 (4.1%) formerly democratic enterprises had degenerated into a state without employee-ownership by the time of the study (see above). Overall, in most of the cases, the majority of the employees participated in collective ownership of their respective enterprise, which allowed them to participate in strategic and tactical decisions, either directly or indirectly *via* electing their representatives into the democratic boards.

### Research question 1: Conditions, practices, and psychological phenomena contributing to degeneration

Research question 1 aimed to identify detrimental organizational and external conditions, non-democratic or non-participative practices, and psychological phenomena that contribute to the degeneration of an enterprise. For a better overview we differentiated between all forms of *full degeneration* (Table 2) and all forms of *degeneration tendency* (Table 3).

**Table 2:** Frequencies of conditions, practices, and psychological phenomena of *all forms of full degeneration* mentioned (*n* = 7 enterprises).

**Conditions, practices, and psychological phenomena**	**Frequency**	**References**
1.**Organizational/external conditions:**	**15**	
Market demands/pressure/competition	4	Lindenfeld, 2001; Bittencourt Meira, 2014; Narvaiza et al., 2017; Borowiak and Ji, 2019
Financial crisis	1	Narvaiza et al., 2017
Forces of external stakeholders (bank, customer)	3	Bittencourt Meira, 2014; Kranz and Steger, 2016; Narvaiza et al., 2017
Insufficient support through external expertise	2	Bittencourt Meira, 2014; Narvaiza et al., 2017
Passivity of union/ambivalent union relations	2	Kalmi, 2002; Borowiak and Ji, 2019
Wrong business decisions	1	Sacchetti and Tortia, 2020
Issued more shares for liquidity/worker owner expectations not fulfilled	1	Lindenfeld, 2001
Product quality problems	1	Bittencourt Meira, 2014
2. **Non-democratic or non-participative practices:**	**11**	
**2.1 Exclusion**		
Excluding new employees from ownership	2	Kalmi, 2002; Bittencourt Meira, 2014
**2.2 Dysfunctional Human Resources management**		
No/insufficient cooperative education and training	1	Kalmi, 2002
**2.3 Deficient cooperative culture and climate**		
Shaping a culture of involvement and deliberation failed/unfamiliarity with employee ownership concept	2	Kranz and Steger, 2016; Sacchetti and Tortia, 2020
Competitive climate against external competitors	1	Borowiak and Ji, 2019
Intraorganizational conflicts	2	Lindenfeld, 2001; Narvaiza et al., 2017
**2.4 Neglect of participation**		
Goals of employee ownership were not democratizing but dividends and secure employment	1	Kalmi, 2002
Organization of labor: Low participation opportunities in decision-making	2	Kalmi, 2002; Borowiak and Ji, 2019
3. **Psychological phenomena:**	**4**	
Weak initial commitment to employee ownership/forced choice of employee buy out	3	Lindenfeld, 2001; Kalmi, 2002; Kranz and Steger, 2016
Low employee commitment to the cooperative idea/low willingness to participate	1	Borowiak and Ji, 2019

**Table 3:** Frequencies of conditions, practices, and psychological phenomena of *all forms of degeneration* **tendency** mentioned (*n* = 20 enterprises).

**Conditions, practices, and psychological phenomena**	**Frequency**	**References**
**1. Organizational/external conditions:**	**20**	
Market demands/pressure/competition	10	Schoening, 2006; Jensen, 2011 (5 enterprises); Vieta, 2012; Cathcart, 2014; Meyers and Vallas, 2016; Pansera and Rizzi, 2020
Size of the company (growth)/expansion	6	Meyers, 2006, 2011; Schoening, 2006; Kennelly and Odekon, 2016; Meyers and Vallas, 2016; Hoffman and Shipper, 2018; Pansera and Rizzi, 2020
Crisis (ideological scandal)	1	Meyers, 2006, 2011
Passivity of union/ambivalent union relations	3	Kalmi, 2002 (2 enterprises); Calo and Shipper, 2018
**2. Non-democratic or non-participative practices:**	**27**	
**2.1 Exclusion**		
Excluding new employees from ownership	9	Kalmi, 2002 (3 enterprises); Jensen, 2011 (5 enterprises); Pansera and Rizzi, 2020
**2.2 Dysfunctional human resources management**		
No / insufficient cooperative education and training	4	Kalmi, 2002 (3 enterprises); Vieta, 2014
**2.3 Deficient cooperative culture and climate**		
Competitive climate against external competitors	1	Calo and Shipper, 2018
Economistic managerial rhetoric	1	Cathcart, 2014
Intraorganizational conflicts	2	Darr, 1999; Cathcart, 2014
Fostering inequality	4	Jensen, 2011 (3 enterprises); Cathcart, 2014
**2.4 Neglect of participation**		
Goals of employee ownership were not democratizing but dividends and secure employment	4	Kalmi, 2002 (3 enterprises); Cathcart, 2014
Organization of labor: Low participation opportunities in decision-making	2	Kalmi, 2002 (2 enterprises)
**3. Psychological phenomena:**	**10**	
Weak initial commitment to employee ownership	3	Kalmi, 2002 (3 enterprises)
Perceived weak organizational justice	1	Darr, 1999
High praise of the director prevents redemocratization	1	Jochmann-Döll and Wächter, 2008
Managers caused a breach of trust	1	Meyers, 2011
Managers use democratic organs as career instrument	1	Cathcart, 2013
Work/performance pressure, stress	1	Calo and Shipper, 2018
Job/organizational dissatisfaction	1	Berry and Schneider, 2011
Strong job satisfaction prevents the formation of democratic work teams	1	Jochmann-Döll and Wächter, 2008

#### Full degeneration

Out of 74 studied enterprises, only seven were classified as *full degeneration*. Table 2 provides the organizational and external conditions, non-democratic and non-participative practices, and psychological phenomena found in those fully degenerated enterprises.

##### Organizational and external conditions

In more than half of the cases, fully degenerated enterprises were faced with strong and changing market conditions, including market deregulation measures (Ji, 2018; Borowiak and Ji, 2019) or distrust of customers toward democratic enterprises (Bittencourt Meira, 2014) that caused decreases in sales (Narvaiza et al., 2017), forcing them to reduce the workforce (Lindenfeld, 2001). Three enterprises received pressure from external stakeholders, in the sense of being dependent on one single bank influencing their financial (Kranz and Steger, 2016) and personnel decisions (Narvaiza et al., 2017). Further mentioned were union passivity in ownership-related matters (Kalmi, 2002), ambivalent relations of employee-owners toward the union that had supported the foundation of the cooperative (Ji, 2018), and ineffective support through external business consultants not familiar with democratic enterprises (Bittencourt Meira, 2014; Narvaiza et al., 2017). Product quality problems (Bittencourt Meira, 2014), making wrong business decisions with respect to expansion (Sacchetti and Tortia, 2020), and issuing additional shares to raise cash for modernization, which meant that worker-owners received reduced benefits and were disappointed by the ownership-model (Lindenfeld, 2001), represented additional detrimental organizational conditions.

##### Non-democratic or non-participative practices

Concerning non-democratic or non-participative practices, fully degenerated enterprises excluded new employees from being owners (Kalmi, 2002; Bittencourt Meira, 2014) and worker-owners were not offered trainings regarding their rights and responsibilities (Kalmi, 2002). Subsequently, such enterprises failed in shaping a culture of involvement and deliberation that could turn unfamiliarity with the worker-ownership concept into something familiar and trustable (Kranz and Steger, 2016; Sacchetti and Tortia, 2020). Additionally, ruthless business practices applied by competing transportation network companies strongly harmed the climate within a taxi cooperative (Borowiak and Ji, 2019). Further, conflicts between management and hourly workers arose due to unequal power relations. Hourly workers typically needed to work harder, were not as respected, and did not wield comparable power and authority, but received lower wages than managers (Lindenfeld, 2001; see also Narvaiza et al., 2017). Obviously, maintaining such inequalities undermines a cooperative culture and climate. Moreover, the organization of labor in two fully degenerated companies was characterized by low opportunities for participation in the daily work, and preconditions for effective participation were not addressed (Kalmi, 2002; Borowiak and Ji, 2019). In addition, the aim of employee ownership was not predominantly focused on democratizing labor relations, but on providing dividends and secure employment (only) for the employee-owners (Kalmi, 2002).

##### Psychological phenomena

Psychological phenomena found in fully degenerated enterprises mainly referred to employees' weak commitment to employee ownership and to the cooperative idea. Due to a forced choice of “taking over the enterprise or losing the job”, initial commitment of workers to employee ownership was weak, as case studies from Estonia, Germany, and Canada indicate (Lindenfeld, 2001; Kalmi, 2002; Kranz and Steger, 2016).

#### Degeneration tendency

Compared to enterprises with full degeneration (*n* = 7), far more enterprises (*n* = 20) were attested only a *degeneration tendency* (see Table 3).

##### Organizational and external conditions

The most frequent organizational/external conditions found to threaten democratic enterprises were unfavorable market demands, pressure and competition in half of the cases (e.g., see Jensen, 2011; Vieta, 2012; Cathcart, 2014). Expansion and enlarging the workforce were also common features limiting organizational democracy. However, growing in size is often an indicator of prosperous organizations. This is also true for democratic enterprises. Due to our definition of constitutional and organizational degeneration tendencies, enterprises can be allocated to this category even if they flourish. For example, the cooperative Home Care Associates started as a small basic-democratic collective with 12 employees, 100% employee-owned. As a result of their success, the number of employees rose to about 2,300 and about 70% of them were worker-owners (Berry and Schneider, 2011; Kennelly and Odekon, 2016). However, since the percentage of worker-owners decreased, this case was categorized as a moderate constitutional degeneration tendency (cf. several publications by Meyers). Finally, passivity of union members in ownership-related matters (Kalmi, 2002), ambivalent relations between worker owners and unions (Calo and Shipper, 2018), and an ideological scandal in one enterprise, were identified as other organizational issues that can foster degenerative tendencies.

##### Non-democratic or non-participative practices

Reviewed studies provide several examples of non-democratic or non-participative practices contributing to degeneration tendencies. Nine enterprises reported that tensions in the workforce existed concerning the issue of exclusion of new employees from ownership. Some argued that it was too expensive for workers to buy in, or that not all workers would want to become owners and assume associated responsibilities, or that new members would not share the values of the founding members (Jensen, 2011). However, especially in the Estonian employee-owned enterprises, it was the management team that did not encourage new employees to acquire ownership, because managers were reluctant to enlarge the number of owners (Kalmi, 2002). In three of the Spanish Sociedades Laborales (SAL) described by Jensen (2011), goal/cultural degeneration tendencies occurred, since these enterprises differentiated between members and non-members when paying the annual bonus. This non-democratic practice undermines cooperative culture by fostering inequality and tensions between cooperative attitudes of solidarity and the self-interest of the respective group of employees (see also Darr, 1999; Jensen, 2011). In some enterprises, missing cooperative education and training (Kalmi, 2002), low opportunities for and inefficient participation in decision-making (Kalmi, 2002) as well as concerns with dividends and secure employment for the employee-owners, instead of democratizing labor relations, as the primary goals of employee ownership, contributed to degenerative tendencies (Kalmi, 2002; Cathcart, 2013).

##### Psychological phenomena

With respect to psychological phenomena that contribute to degenerative tendencies, Kalmi (2002) observed weak initial commitment to employee ownership in three Estonian enterprises. A forced choice between an employee buy-out and losing one's job may explain this weak initial commitment. At Opel Hoppmann, a large German automobile trade (Jochmann-Döll and Wächter, 2008), high praise of the director and high job satisfaction among employees may be responsible for an organizational degeneration tendency. These authors explained that the collective image of the director as a highly competent and “perfect” person permitted employees to justify their own shortcomings and lack of initiatives. Furthermore, due to high job satisfaction, employees felt less compelled to get involved in activities like work teams. Accordingly, Jochmann-Döll and Wächter (2008) reported that work teams, which are one pillar of the democratic model as conceived by the founder, were practically non-existent. In one enterprise, the People's Daily Bread Bakery (California) internal struggles with accountability led to a breach of trust in the democratic founding principles. Instead, a managerial system was established based on representative government rights (Meyers, 2011), indicating an organizational degeneration tendency. Finally, employee-owners' perception that some managers instrumentalized democratic boards for their individual career, may have contributed to a tendency of organizational degeneration (Cathcart, 2013).

### Research question 2: Conditions, practices, and psychological phenomena contributing to retention

Research question 2 referred to the organizational conditions, democratic or participative practices, and psychological phenomena that contribute to the *retention* of organizational democracy (see Table 4). As reported above, the majority of the investigated 74 enterprises, precisely 39 (52.7%), resisted degeneration and degenerative tendencies and were classified as retention. In his context, we found several beneficial conditions and practices previously reported by other scholars (e.g., Rothschild-Whitt, 1976; Cornforth, 1995; Bretos et al., 2020), but also identified several practices not discussed elsewhere.

**Table 4:** Frequencies of conditions, practices, and psychological phenomena of *all forms of retention* mentioned (*n* = 39 enterprises).

**Conditions, practices, and psychological phenomena**	**Frequency**	**References**
**1. Organizational/external conditions:**	**14**	
Small size of the company	6	Jackall, 1984; Cornforth, 1995 (2 enterprises); Ng and Ng, 2009 (3 enterprises);
Stable consumer base/market, access to a strong market	2	Majee and Hoyt, 2009; Gupta, 2014
Low capital intensity	1	Sacchetti and Tortia, 2020
Low complexity of the production processes	2	Greenberg, 1980, 1984
Support through union/positive union relations	2	Timur and Timur, 2016; Dai et al., 2019
Industry: strong worker identity with their profession as deep miners	1	Hoffmann, 2006
**2. Democratic or participative practices:**	**181**	
**2.1 Integration and delimitation (boundary management)**		
Linking with broader social movements to promote community-based goals	21	Hadley and Goldsmith, 1995; Ng and Ng, 2009; Majee and Hoyt, 2010; Cornwell, 2012; Vieta, 2012; 2014 (3 enterprises); Gupta, 2014; Langmead, 2016, 2017; Sobering, 2016; Timur and Timur, 2016; Hoffman and Shipper, 2018 (2 enterprises); Dai et al., 2019; Westoby and Shevellar, 2019; Wren, 2020 (3 enterprises); Kociatkiewicz et al., 2021 (2 enterprises)
Effort to stay small/resist to grow	2	Gupta, 2014; Jaumier, 2017
**2.2 Value-based human resources management**		
Value-based personnel selection	11	Cornforth, 1995; Hoffmann, 2001, 2003, 2006; Majee and Hoyt, 2009; Gupta, 2014; Hoffman and Shipper, 2018 (2 enterprises); Wren, 2020 (3 enterprises)
Common recruitment and induction procedures	13	Jackall, 1984; Hoffmann, 2003, 2006; Timur and Timur, 2016; Langmead, 2017 (2 enterprises); Hoffman and Shipper, 2018 (2 enterprises); Wren, 2020 (3 enterprises); Kociatkiewicz et al., 2021
Cooperative education and training	18	Hoffmann, 2003; Ng and Ng, 2009 (3 enterprises); Majee and Hoyt, 2010; Cornwell, 2012; Vieta, 2012; 2014 (3 enterprises); Ashforth and Reingen, 2014; Boguslaw and Taghvai-Soroui, 2018; Hoffman and Shipper, 2018 (2 enterprises); Sobering, 2019; Wren, 2020 (3 enterprises); Kociatkiewicz et al., 2021
Further value-based socialization process	16	Jackall, 1984; Hoffmann, 2001, 2003; Atzeni and Ghigliani, 2007; Majee and Hoyt, 2010; Cornwell, 2012; Vieta, 2012; 2014 (3 enterprises); Langmead, 2016, 2017; Timur and Timur, 2016; Hoffman and Shipper, 2018; Wren, 2020 (3 enterprises)
Knowledge/information/experience sharing	15	Greenberg, 1980, 1984 (3 enterprises); Cornforth, 1995 (2 enterprises); Ng and Ng, 2009 (3 enterprises); Majee and Hoyt, 2010; Langmead, 2016, 2017; Hoffman and Shipper, 2018 (3 enterprises); Sobering, 2019
Job rotation/job enlargement/job enrichment (production, service or administrative tasks)	15	Greenberg, 1980, 1984 (2 enterprises); Cornforth, 1995 (2 enterprises); Ng and Ng, 2009; Jensen, 2011; Gupta, 2014; Sobering, 2016; Langmead, 2017; Harrison et al., 2018; Wren, 2020 (2 enterprises); Kociatkiewicz et al., 2021 (2 enterprises)
**2.3 Support of cooperative culture and climate**		
Open criticism and discussion, permanent requirement for accountability and overt critique of managers or elected representatives	14	Greenberg, 1980, 1984 (2 enterprises); Hoffmann, 2005, 2006 (2 enterprises); Ng and Ng, 2009; Ashforth and Reingen, 2014; Langmead, 2016, 2017; Jaumier, 2017; Sobering, 2019; Kociatkiewicz et al., 2021 (2 enterprises)
Social discourses emphasizing democracy, social transformation, and community development	7	Majee and Hoyt, 2009; Vieta, 2012, 2014 (3 enterprises); Ashforth and Reingen, 2014; Sobering, 2016; Hoffman and Shipper, 2018
Fostering equality	20	Greenberg, 1980, 1984 (2 enterprises); Hoffmann, 2006; Jensen, 2011; Cornwell, 2012; Vieta, 2012; 2014 (3 enterprises); Gupta, 2014; Langmead, 2016, 2017; Sobering, 2016, 2019; Jaumier, 2017; Harrison et al., 2018; Hoffman and Shipper, 2018 (2 enterprises); Dai et al., 2019; Wren, 2020 (2 enterprises); Kociatkiewicz et al., 2021
Dispute resolution system	3	Hoffmann, 2001, 2005; Ashforth and Reingen, 2014
**2.4 Support of participation**		
Random selection or frequent change of democratic board members	4	Greenberg, 1980, 1984 (3 enterprises); Hoffmann, 2005
Democratic leadership/peer monitoring/weak span of control	5	Greenberg, 1980, 1984 (3 enterprises); Atzeni and Ghigliani, 2007; Jensen, 2011
Organization of labor: reinforcement of broad-based participation	17	Greenberg, 1984 (2 enterprises); Hadley and Goldsmith, 1995; Majee and Hoyt, 2010; Jensen, 2011; Cornwell, 2012; Gupta, 2014; Langmead, 2016, 2017; Jaumier, 2017; Boguslaw and Taghvai-Soroui, 2018 Harrison et al., 2018; Hoffman and Shipper, 2018; Sobering, 2019; Sacchetti and Tortia, 2020; Kociatkiewicz et al., 2021 (2 enterprises)
**3. Psychological phenomena:**	**78**	
Strong commitment to cooperative idea and to the enterprise/loyalty/“family”	24	Greenberg, 1984 (2 enterprises); Hadley and Goldsmith, 1995; Hoffmann, 2005, 2006; Ng and Ng, 2009 (3 enterprises); Jensen, 2011; Cornwell, 2012; Ashforth and Reingen, 2014; Gupta, 2014; Langmead, 2016, 2017; Sobering, 2016; Timur and Timur, 2016; Harrison et al., 2018; Hoffman and Shipper, 2018; Dai et al., 2019; Wren, 2020 (3 enterprises); Kociatkiewicz et al., 2021 (2 enterprises)
Psychological ownership	17	Greenberg, 1984 (2 enterprises); Ng and Ng, 2009 (2 enterprises); Hoffmann, 2016 (2 enterprises); Timur and Timur, 2016; Langmead, 2017 (2 enterprises); Harrison et al., 2018; Hoffman and Shipper, 2018 (2 enterprises); Wren, 2020 (3 enterprises); Kociatkiewicz et al., 2021 (2 enterprises)
Generativity	1	Langmead, 2017
Perceived organizational justice	3	Hoffmann, 2001, 2005; Cornwell, 2012
Mutual perspective taking and care/prosociality	15	Greenberg, 1980, 1984 (3 enterprises); Jackall, 1984; Vieta, 2012; 2014 (3 enterprises); Ashforth and Reingen, 2014; Hoffmann, 2016 (4 enterprises); Harrison et al., 2018; Dai et al., 2019; Westoby and Shevellar, 2019
Social inclusion	4	Sobering, 2016; Westoby and Shevellar, 2019; Kociatkiewicz et al., 2021 (2 enterprises)
Sacrifices for the organizational community	9	Oliver, 1984; Gupta, 2014; Langmead, 2016, 2017; Boguslaw and Taghvai-Soroui, 2018; Hoffman and Shipper, 2018 (3 enterprises); Kociatkiewicz et al., 2021
Entrepreneurial motives/readiness for innovative behavior	5	Greenberg, 1980, 1984 (3 enterprises); Langmead, 2016, 2017
Job/organizational satisfaction	1	Dai et al., 2019

#### Organizational and external conditions

Organizational and external conditions were relatively sparsely reported. Small company size was mentioned in six cases as a supportive condition to maintain basic democratic practices, facilitating direct participation in decision-making (Cornforth, 1995; Ng and Ng, 2009; Gupta, 2014; Jaumier, 2017), which is also the case for low complexity of the production process. Further, apparently, a stable market and consumer base can support the economic survival of democratic enterprises. Finally, some industries might be better suited for sustaining democratic principles. For example, deep miners showed a strong worker identity and thus also hold strong commitments to their Welsh cooperative colliery (Hoffmann, 2006), which is an important prerequisite for the retention of democratic structures.

#### Democratic or participative practices

Democratic and participative practices contributing to retention are mentioned in numerous instances. Linking with broader social movements to promote community-based goals is a practice that has been suggested before (Rothschild-Whitt, 1976; Diamantopoulos, 2012; Bretos et al., 2020). In the present systematic review, 21 successful, i.e., not degenerated, democratic enterprises showcased different ways of connecting with social movements and the community. For example, W. L. Gore and Associates permits every employee 8–16 paid hours per year to work with non-profits in their community (Hoffman and Shipper, 2018). The Cheeseboard Collective (a bakery and restaurant in USA) donates money and in-kind products to local public schools, gives away food to homeless shelters, and strengthens the cooperative movement by sending their members to workshops and conferences, creating networks, and encouraging schools to visit them to see how a cooperative operates (Gupta, 2014; for cases from different contexts see, e.g., Hadley and Goldsmith, 1995; Majee and Hoyt, 2009, 2010; Vieta, 2012; 2014; Kociatkiewicz et al., 2021). Furthermore, the Cheese Board Collective and Scopex (a shaping of sheet firm in France) put effort into consciously staying small in order not to endanger their basic democratic principles and practices (Gupta, 2014; Jaumier, 2017).

Applying methods of Human Resources Management (HRM) that are based on cooperative and democratic values, 18 enterprises that resisted degeneration and degenerative tendencies indicated that they continuously educate employees on cooperative values and train them in new skills (e.g., see Hoffmann, 2003; Hoffman and Shipper, 2018; Wren, 2020). Thus, workers acquire knowledge about entrepreneurial tasks, participation rights, and responsibilities. Moreover, democratic and cooperative principles can be internalized by introducing or connecting new and older members (Ng and Ng, 2009). Value-based employee selection, common recruitment and induction procedures, as well as further socialization processes are frequently used participative practices that consolidated the retention of democratic enterprise structures. Democratic enterprises put a lot of effort into the recruitment and subsequent socialization process: first they make sure that the applicants' values are aligned with the company's democratic and cooperative ideals; second, the applicant has to pass a probation period of working in different areas to get to know as many members as possible; and, thirdly, the decision for or against hiring a new member is based on consensus of all members or a membership committee. Through mentoring programs, newcomers are introduced into the company and trained in cooperative practices through collaborative and informal learning (e.g., Vieta, 2012, 2014; Wren, 2020). Information-, knowledge-, and experience-sharing as well as personality-promoting forms of working (like job rotation between production, service, and administrative tasks; job enlargement; job enrichment) are further important participative practices that many enterprises used for resisting degeneration (e.g., see Oliver, 1984; Cornforth, 1995; Jensen, 2011; Harrison et al., 2018; Hoffman and Shipper, 2018). These practices include, for example, an open book and open-door policy, newsletters, annual reports, and the publication and discussion of minutes of board meetings (Greenberg, 1984; Majee and Hoyt, 2009). Information transfer seems to be most effective if board members hold regular jobs and disseminate information to their colleagues on the shop floor as it was the case in the Pacific-Northwest plywood cooperatives in the USA (Greenberg, 1984).

Supporting the development of a cooperative culture and work climate, e.g., through fostering equality among employees, was a frequently applied practice realized in 20 cases. For instance, this was achieved by distributing the annual bonus equally among members and non-members (Jensen, 2011), exercising the one person-one-vote principle, upholding similar rights for old and new members, and equal pay (Jackall, 1984) or at least limited wage differentials (Jaumier, 2017). Open criticism and discussion as well as accountability and overt scrutiny of managers are further democratic practices mentioned frequently as contributing to democratic retention (e.g., see Greenberg, 1980, 1984; Jackall, 1984; Kociatkiewicz et al., 2021). In respective enterprises, conflicts were not suppressed (Ng and Ng, 2009; Ashforth and Reingen, 2014), official grievance and dispute resolution procedures were implemented and frequently used (Hoffmann, 2001, 2006; Ashforth and Reingen, 2014), and members refused the division into those who command and those who obey—adhering to an attitude of “we are all bosses” instead (Jaumier, 2017). In Natura, a natural foods co-op in a large US city, conflicts are an integral component of their democratic functioning (Ashforth and Reingen, 2014). By advocating and practicing rituals “… the messiness of managing the duality was held somewhat in check by regularized calls before meetings for keeping conflict in bounds, regularized interventions during meetings when conflict went out of bounds, and regularized compliments and thank-yous to opponents after contentious meetings” (Ashforth and Reingen, 2014, p. 505). Another democratic practice was that, members were encouraged to engage in ongoing social discourses on democracy, social transformation, and community development as it was common in Argentinian worker recuperated enterprises (Vieta, 2012, 2014). Similarly, in the Caring Home Services cooperative (Majee and Hoyt, 2009), values like fairness, equity, and respect were constantly reviewed, updated, and reinforced to secure that business operations and workers' actions were aligned with these principles.

The organization of labor plays a crucial and frequently mentioned role in the socialization process by supporting broad-based participation. In smaller enterprises, consensus-based decision-making is common to make sure that diverse opinions and viewpoints are taken seriously, leading to an ongoing negotiation of social reality (e.g., Oliver, 1984). In bigger enterprises, like the Scott Bader Commonwealth (Hadley and Goldsmith, 1995) or the Plywood Cooperatives (Greenberg, 1984), both direct and representative forms of participation were practiced in each case. Members typically participated directly in the assembly and voted for representatives in boards or councils with regular rotations (e.g., Cornforth, 1995; Jensen, 2011). In a few cases, members of boards were randomly selected or frequently rotated to prevent organizational degeneration (e.g., Greenberg, 1980; Hoffmann, 2005). In this vein, members maintained control of the enterprise, and, as Greenberg (1984) summarized: “… direct and representative democracy can not only exist together but can also serve to enrich each other” (p. 213). In five cases, democratic or self-leadership was practiced, creating broad spans of control and allowing employees more autonomy in their daily work (e.g., Greenberg, 1980; Atzeni and Ghigliani, 2007; Jensen, 2011).

#### Psychological phenomena

Several psychological phenomena that may foster the retention of democratic enterprises could be identified. First, 24 companies indicated a strong commitment to the cooperative idea and the enterprise. Member employees are convinced of the founding principles of democracy, solidarity, and equality and, due to constructively resolving conflicts, feel a strong bonding among members, experiencing the collective as a family unit (e.g., Ng and Ng, 2009; Gupta, 2014; Harrison et al., 2018). A second psychological phenomena, found in 17 democratic enterprises resisting degeneration, is strong psychological ownership experienced by working members, e.g., see the ethnographic study on two Polish co-operatives practicing solidarity economy by Kociatkiewicz et al. (2021; cf. also studies by Hoffmann, 2016; Wren, 2020). Pierce et al. (2001) defined psychological ownership as an affective and cognitive state of mind “in which individuals feel as though the target of ownership (material or immaterial in nature) or a piece of it is ‘theirs'. The core of psychological ownership is the feeling of responsibility and being psychologically tied to an object” (Pierce et al., 2001, p. 299). Through possessing shares, employees are real owners and have a strong sense that it is them who run the enterprise. Accordingly, they feel responsible and accountable for their actions, developing an identity of ownership (Greenberg, 1984; Hoffman and Shipper, 2018). In connection with this ownership identity, in nine enterprises, employees were willing to accept personal sacrifice for the success and survival of the community; e.g., accepting pay cuts and unpaid overtime before considering leaving (Oliver, 1984; Hoffman and Shipper, 2018), and 15 companies emphasized their employees' mutual perspective taking, caring for and helping out colleagues if necessary (e.g., see Greenberg, 1980, 1984; Harrison et al., 2018; Dai et al., 2019; Westoby and Shevellar, 2019).

### Research question 3: Conditions, practices, and psychological phenomena contributing to regeneration

Research question 3 asked what organizational/external conditions, democratic or participative practices, and psychological phenomena contribute to the regeneration of an enterprise (see Table 5). Overall, we identified eight democratic enterprises as fulfilling conditions for *regeneration* (10.8%).

**Table 5:** Frequencies of conditions, practices and psychological phenomena of **all forms of regeneration** mentioned (*n* = 8 enterprises).

**Conditions, practices, and psychological phenomena**	**Frequency**	**References**
**1. Organizational/external conditions:**	**0**	
**2. Democratic or participative practices:**	**21**	
**2.1 Integration and delimitation (boundary management)**		
Linking with broader social movements to promote community-based goals	3	Bryer, 2011; Narvaiza et al., 2017 (2 enterprises)
Effort to stay small/resist to grow/cell division	2	Cornforth, 1995 (2 enterprises)
Support through external expertise	1	Narvaiza et al., 2017
**2.2 Value-based human resources management**		
Common recruitment and induction procedures	1	Kennelly and Odekon, 2016
Cooperative education and training	2	Varman and Chakrabarti, 2004; Narvaiza et al., 2017
Further value-based socialization process	1	Varman and Chakrabarti, 2004
Knowledge/information/experience sharing to rebuilt trust	2	Jones, 2000; Kandathil and Varman, 2007
Job rotation/job enlargement/job enrichment (production, service or administrative tasks)	1	Cornforth, 1995
**2.3 Support of cooperative culture and climate**		
Fostering equality	2	Varman and Chakrabarti, 2004; Kandathil and Varman, 2007
Open criticism and discussion, permanent requirement for accountability and overt critique of managers or elected representatives	1	Bryer, 2011
Social discourses emphasizing democracy, social transformation, and community development	1	Bryer, 2011
**2.4 Support of participation**		
Organization of labor: reinforcement of broad-based participation	4	Jones, 2000; Varman and Chakrabarti, 2004; Kennelly and Odekon, 2016; Narvaiza et al., 2017
**3. Psychological phenomena:**	**4**	
Strong commitment to cooperative idea and to the enterprise/loyalty/“family”	3	Bryer, 2011; Kennelly and Odekon, 2016; Narvaiza et al., 2017
Sacrifices for the organizational community	1	Bryer, 2011

#### Organizational and external conditions

Concerning the regenerated enterprises, the studies reported no organizational or external conditions that were helpful in the regeneration process.

#### Democratic or participative practices

With respect to democratic or participative practices that contribute to regeneration, we found fewer but similar aspects as for retention (presented in Section Research question 2: Conditions, practices, and psychological phenomena contributing to retention).

In three enterprises, a small Spanish baking equipment firm, a medium-sized French construction cooperative, and an Argentinean recuperated cooperative, links with broader social movements, namely a network of companioned worker cooperatives and a business ethics network, respectively, supported their goal and cultural regeneration process (see Bryer, 2011; Narvaiza et al., 2017). Two other small worker cooperatives countered organizational degeneration when growing larger through segmentation in basic-democratic divisions (Cornforth, 1995).

Knowledge-, information-, or experience- sharing were reported as participative practices in two enterprises that had degenerated due to unequal treatment of members and mistrust in management. Both enterprises were thus able to rebuild trust in management. In one enterprise, a small British wholesaler cooperative, the management committee, which was elected by the workers, regularly reported their activities, including presenting and discussing the business plan, installing a weekly discussion for current issues, and making available meeting minutes for all members to scrutinize (Jones, 2000). In the other enterprise, a big Indian jute mills, the new president held a series of meetings with all employees, made himself accessible, and asked for employee opinions and suggestions *via* installing complaint boxes. In combination with treating all employees equally, e.g., through consistent and fair rules, the new president achieved to rebuild trust in management and contributed to the improvement of the cooperative climate (Kandathil and Varman, 2007). As mentioned above, fostering equality by electing unskilled workers as representatives was a rather unusual democratic practice implemented in a medium-sized Indian service cooperative to facilitate the regenerating process (Varman and Chakrabarti, 2004). As an example, for value-based HRM, cooperative education and training was another supporting democratic practice. Unskilled workers were empowered by skill development and enhanced democratic consciousness. This socialization process as a democratic practice for fostering regeneration resulted in the implementation of a culture of equality (Varman and Chakrabarti, 2004). Within a recuperated cooperative in Argentina open criticism and discussion were important ingredients in ongoing dialogues between individual and collective needs as well as about the purposes of their activity within society. Hence, social discourses emphasizing democracy, social transformation and community development were psychological practices that supported the cooperative to regenerate from their degenerating tendencies (Bryer, 2011). Further, HRM practices that helped in the regeneration process were common recruitment and induction procedures. Applicants had longer probationary periods, were mostly local workers, and consensual decisions were made after getting feedback from the whole staff (Kennelly and Odekon, 2016).

The organization of labor and the reinforcement of broad-based participation were reported as democratic practices fostering regeneration in four of the eight cases. In two enterprises (Jones, 2000; Kennelly and Odekon, 2016), direct elements of participation were re-introduced (e.g., consensual decisions and general meetings). In the third enterprise, unskilled workers took the initiative in the general assembly until all elected positions were held by these low skilled employees (Varman and Chakrabarti, 2004). In the fourth company, the strongly hierarchical organizational structure was replaced by a horizontal structure focusing on democratic work groups whose members elected their leaders (Narvaiza et al., 2017).

#### Psychological phenomena

We identified only two psychological phenomena that contributed to enterprises' regeneration. Three enterprises reported employees' strong commitment to the cooperative idea and to the enterprise (Bryer, 2011; Kennelly and Odekon, 2016; Narvaiza et al., 2017). Additionally, in an Argentinean recuperated cooperative, members' willingness to accept personal sacrifices for the organizational community was one psychological phenomenon considered vital to their regeneration process (Bryer, 2011).

### Research question 4: The Mondragon Cooperative Cooperation network and its forms of transformation as well as relating conditions, practices, and psychological phenomena

In 2019, the Mondragon Cooperative Corporation (MCC) encompassed about 264 firms, 98 of them worker cooperatives, and had 81,837 employees. It was divided into an industrial, a retail, a service, and an educational sector (see, also in the following, Basterretxea et al., 2022). In 2020, 75.0% of employees in the industrial sector were cooperative members (https://www.mondragon-corporation.com/urtekotxostena/datos-basicos.php?l=en; retrieved April 19, 2022). In the following, a multi-national worker cooperative that comprises a parent cooperative and several subsidiaries is termed an *enterprise group*.

Table 6 shows an overview of all nine cases of enterprises or enterprise groups within the Basque Mondragon network, which met inclusion criteria based on methodological standards. Overall, the included studies contain only one democratic enterprise group, namely the ULMA group, which represented a case of *retention* at the time of investigation. Further, three other enterprises (Fagor Electrodomésticos Group, LANA cooperative, MAPSA cooperative) exhibited *degeneration tendencies* without any successful attempts of regeneration reported in the available studies. One of these, the Fagor Electrodomésticos group—which some years prior was the largest industrial worker cooperative in the world—closed in 2013 because of insolvency after 57 years of mostly successful democratic and economic activity (Basterretxea et al., 2019). Five democratic enterprises (Coprecci cooperative, EB cooperative, Eroski group, Fagor Ederlan group, Maier group) underwent processes of *regeneration*, although three of those still showed moderate (Coprecci cooperative, Fagor Ederlan group) or stronger *tendencies* (Eroski group) of a fragmentary form of *degeneration* during the investigation period (cf. the conclusions regarding an “ongoing tension” of degenerative and regenerative processes by Storey et al., 2014; Bretos et al., 2020).

**Table 6:** Number of enterprise cases per transformation form from the Mondragon Cooperatives network.

**Degeneration tendency (number of enterprises)** ***n*** = **3**		
**Goal/cultural degeneration tendency**	**Moderate Constitutional degeneration tendency (worker owners = majority) AND Goal/cultural degeneration tendency**	**Strong Constitutional degeneration tendency (worker owners = minority) AND goal/cultural degeneration tendency**
MAPSA cooperative (Cheney, 1997, 2001)	LANA cooperative/part of EREIN group (Taylor, 1994)	Fagor Electrodomésticos Group Bankrupt in 2013 (Errasti et al., 2016, 2017; Bretos and Errasti, 2018; Basterretxea et al., 2019, 2022; Bretos et al., 2019)
**Degeneration tendency AND regeneration (number of enterprises)** ***n*** **= 3**		
**Moderate constitutional degeneration tendency (worker owners = majority) AND goal/cultural regeneration**	**Strong constitutional degeneration tendency (worker owners = minority) AND goal/cultural regeneration**	**Goal/cultural degeneration tendency AND constitutional AND organizational regeneration**
Coprecci cooperative / part of the then FAGOR group (Taylor, 1994)	Eroski group (Flecha and Ngai, 2014; Storey et al., 2014; Basterretxea and Storey, 2018)	Fagor Ederlan group (Bretos and Errasti, 2017, 2018; Bretos et al., 2018)
**Retention (number of enterprises)** ***n*** **= 1 (Longitudinal information)**		
ULMA group (Cheney, 1997, 2001; Flecha and Ngai, 2014; left MCC temporarily in 1992)		
**Regeneration (number of enterprises)** ***n*** **= 2**		
**Constitutional AND goal/cultural regeneration**	**Organizational and goal/cultural regeneration**	
Maier group (Flecha and Ngai, 2014)	EB cooperative (anonymized) (Narvaiza et al., 2017)	

To evaluate the state of organizational democracy it is relevant whether the majority of employees was participating in democratic governance of their company at the time of investigation. Supplementary Table 5 provides this information. Five of the nine cases represent enterprises in which the majority of workers were owners (Coprecci, EB cooperative, Fagor Ederlan group, LANA, ULMA group), whereas in two enterprises (Fagor Electrodomésticos group, Eroski group) ownership by workers was <50%, but higher than 33%. This is considered a *structural polarization*—representing a structural indicator of constitutional degeneration—between a democratically ruling minority of working members and a majority of salaried workers excluded from democratic decision-making. For two enterprises (Maier group, MAPSA cooperative), the proportion of employee-owners was not specified in the respective studies.

Available studies do not provide a clear-cut picture of transformation tendencies in the overall Mondragon network. However, considering the size and complexity of the included enterprise groups, a closer look at these cases seems warranted.

#### Degeneration tendency

The Fagor Electrodomésticos group (founded in 1956; comprising 10,470 employees at 18 plants in Spain, France, China, Poland, Morocco, and Italy in 2006, but in 2013, the year of closure, only 5,500 employees remained; Bretos et al., 2019) represents an example of MCC multinational industrial cooperatives. Both showed tendencies of strong *constitutional degeneration* through acquiring capitalist subsidiaries in several continents in the mid-2000s. This expansion was partly forced by the capitalist “liberation” of international markets. In contrast to Fagor Ederlan, Fagor Electrodomésticos did not solve the programmatic contradiction between cooperative and capitalist principles: Only 35.4% of its workforce were cooperative members in its last years (Basterretxea et al., 2019), and, finally, it became insolvent after 50 years of economic success. In their grounded-theory based study, Basterretxea et al. (2019, p. 589) summarize the following main reasons for failure as perceived by experienced long-time executives, managers, workers, and other stakeholders:

“The burst of Spanish property bubble and the consequent drop in sales of household appliances, marketing and product positioning problems, the increased bargaining power of big retailers entrants …, the increased competitive rivalry, the poor implementation of the internationalization strategy, the size problem (too small compared to main competitors and too big to be efficiently run as a cooperative), failures in the cooperative governing bodies, and—above all—poor decisions about major investments.”

Further organizational conditions and practices that may have contributed to tendencies of constitutional and goal/cultural degeneration of the enterprise group and, eventually, to its demise, are reported in the respective studies included in the present review (see Table 7 and, in detail, Errasti et al., 2016, 2017; Bretos and Errasti, 2018; Basterretxea et al., 2019, 2022; Bretos et al., 2019), for example: product quality problems, resistance of unions against crises-induced dismissals, inappropriate personnel recruitment and selection (e.g., nepotism resulting in skills gaps and shirking), insufficient cooperative training and transfer of the cooperative values to new workers, lack of trust between parent coops and foreign subsidiaries, lack of cooperative tradition in foreign subsidiaries, reverse dominance hierarchy (basis-democratic governance and stakeholder democracy hampered the implementation of unpopular adaptation measures) and unbalanced power of interest groups leading to a climate of critique and accusation against management, abrupt turnover in the company's top management and councils, deskilling Tayloristic work organization, toleration of absenteeism and shirking through laissez-faire leadership. Psychological phenomena that were associated with degeneration tendencies comprise the lack of expertise of democratic board members and top managers, slow and often difficult decision-making and conflicts in democratic bodies, waiving the inclusion of independent experts into the councils, high work pressure and employee resistance against humanization of work and participative practices, especially in several subsidiaries. In contrast to its foreign subsidiaries, considered by itself, the parent cooperative of Fagor Electrodomésticos did not display any substantive constitutional or organizational degeneration. Cooperative members without any management functions, including both skilled and unskilled workers, technicians, and craftsmen, maintained a strong influence on strategic decisions in the general assembly, the governing council, and the social council (Errasti et al., 2017; Basterretxea et al., 2019). In sharp contrast to most conventional capitalist corporations, the majority of working members of Fagor Electrodomésticos were reallocated to other MCC cooperatives after the closure (Errasti et al., 2017; Arando and Bengoa, 2018).

**Table 7:** Frequencies of conditions, practices, and psychological phenomena of *all forms of degeneration tendency* mentioned (*n* = 6 cases of the Mondragon Cooperative Corporation).

**Conditions, practices, and psychological phenomena**	**Frequency**	**Enterprise groups and publications**
**1. Organizational/external conditions:**	**13**	
Market demands/pressure/competition/no stable consumer base/market	3	Eroski group (Basterretxea and Storey, 2018^*^); Fagor Ederlan group (Bretos and Errasti, 2018^*^; Bretos et al., 2018^*^; Bretos et al., 2019^*^); Fagor Electrodomésticos group (Errasti et al., 2016; Bretos and Errasti, 2018; Basterretxea et al., 2019, 2022; Bretos et al., 2019)
Forces of (semi-external) stakeholders (MCC bank)	2	Coprecci cooperative (Taylor, 1994)^*^; LANA (Taylor, 1994)
Size of the company (growth)/expansion	2	Fagor Ederlan group (Bretos and Errasti, 2018^*^; Bretos et al., 2018^*^); Fagor Electrodomésticos group (Bretos and Errasti, 2018; Basterretxea et al., 2019; Bretos et al., 2019)
Financial crisis	2	Eroski group (Storey et al., 2014^*^; Basterretxea and Storey, 2018^*^); Fagor Electrodomésticos group (Errasti et al., 2016, 2017; Bretos et al., 2019)
Wrong business decisions	1	Fagor Electrodomésticos group (Errasti et al., 2017; Basterretxea et al., 2019, 2022)
Lack of unions/ambivalent union relations/union resistance against bad working conditions	2	Fagor Ederlan group (Bretos and Errasti, 2018^*^; Bretos et al., 2019^*^); Fagor Electrodomésticos group (Errasti et al., 2016; Bretos et al., 2019)
Product quality problems	1	Fagor Electrodomésticos group (Basterretxea et al., 2022)
**2. Non-democratic or non-participative practices:**	**24**	
**2.1 Exclusion and boundary management**		
Missing Cooperativization of capitalist subsidiaries/economistically motivated exclusion of ownership/cooperative members	3	Fagor Ederlan group (Bretos and Errasti, 2018^*^; Bretos et al., 2018^*^; Bretos et al., 2019^*^); Fagor Electrodomésticos group (Errasti et al., 2016; Bretos and Errasti, 2018; Bretos et al., 2019); LANA (Taylor, 1994)
Waiver of external expertise in democratic boards	1	Fagor Electrodomésticos group (Basterretxea et al., 2022)
**2.2 Dysfunctional human resources management**		
Dysfunctional recruitment, selection, and induction procedures	1	Fagor Electrodomésticos group (Basterretxea et al., 2019)
No/insufficient cooperative education and training	2	Fagor Ederlan group (Bretos and Errasti, 2018^*^; Bretos et al., 2018^*^; Bretos et al., 2019^*^); Fagor Electrodomésticos group (Basterretxea et al., 2019; Bretos et al., 2019)
**2.3 Deficient cooperative culture and climate**		
Lack of a collectivistic culture and cooperative tradition in foreign subsidiaries	2	Fagor Ederlan group (Bretos and Errasti, 2018^*^); Fagor Electrodomésticos group (Bretos and Errasti, 2018)
Lack of trust between parent coops and foreign subsidiaries	2	Fagor Ederlan group (Bretos and Errasti, 2018^*^; Bretos et al., 2018^*^; Bretos et al., 2019^*^); Fagor Electrodomésticos group (Errasti et al., 2016; Bretos and Errasti, 2018; Bretos et al., 2019)
No communication among the foreign subsidiaries	1	Fagor Electrodomésticos group (Errasti et al., 2016)
Intraorganizational conflicts	2	Fagor Ederlan group (Bretos and Errasti, 2017^*^); Fagor Electrodomésticos group (Errasti et al., 2017; Basterretxea et al., 2022)
Economistic managerial rhetoric	2	MAPSA (Cheney, 2001); LANA (Taylor, 1994)
Aggressive/offensive requirement for accountability and overt critique of managers	1	Fagor Electrodomésticos group (Basterretxea et al., 2019, 2022)
**2.4 Neglect of participation**		
Goals of employee ownership were not democratization but dividends and secure employment	2	Fagor Ederlan group (Bretos and Errasti, 2018^*^; Bretos et al., 2018^*^; Bretos et al., 2019^*^); Fagor Electrodomésticos group (Errasti et al., 2016; Bretos et al., 2019)
Organization of work: low participation opportunities in decision-making/transfer of management-controlled, economistic HRM practices/Tayloristic division of labor	4	Fagor Ederlan group (Bretos and Errasti, 2018^*^; Bretos et al., 2018^*^; Bretos et al., 2019^*^); Fagor Electrodomésticos group (Bretos and Errasti, 2018; Basterretxea et al., 2019; Bretos et al., 2019); MAPSA (Cheney, 2001)
Laissez-faire leadership	1	Fagor Electrodomésticos group (Basterretxea et al., 2019)
**3. Psychological phenomena:**	**15**	
Lacking skills and knowledge for strategic decisions/slow decision-making in democratic panels	2	Fagor Electrodomésticos group (Errasti et al., 2017; Basterretxea et al., 2022)
Weak commitment to employee ownership/cooperative idea (employees in subsidiaries)	2	Fagor Ederlan group (Bretos et al., 2018^*^); Fagor Electrodomésticos group (Basterretxea et al., 2019)
Lack of employees' psychological ownership	1	Fagor Ederlan group (Bretos et al., 2019^*^)
Work pressure/stress	3	Fagor Ederlan group (Bretos et al., 2019^*^); Fagor Electrodomésticos group (Bretos et al., 2019)
High manager and council representatives turnover	1	Fagor Electrodomésticos group (Basterretxea et al., 2022)
Job/organizational dissatisfaction of worker members	2	Eroski group (Basterretxea and Storey, 2018^*^); Fagor Electrodomésticos group (Basterretxea et al., 2019)
Freeriding/shirking and absenteeism of worker members	2	Eroski group (Basterretxea and Storey, 2018^*^); Fagor Electrodomésticos group (Basterretxea et al., 2019)
Employees' resistance against humanization of work	1	Fagor Electrodomésticos group (Basterretxea et al., 2019; Bretos et al., 2019)
Wage solidarity against sacrifices for the community	1	Fagor Electrodomésticos group (Basterretxea et al., 2019, 2022)

Several studies described the same conditions, practices, and psychological phenomena for the same enterprise. These phenomena were counted only once for each enterprise. Therefore, the number of publications may exceed the number of counted conditions, practices, and psychological phenomena.

* Degenerative tendency and regeneration activity appear simultaneously in this case.

Additionally, several studies report three—empirically interrelated—indicators of goal/cultural degeneration tendencies within some MCC cooperatives. The first one refers to the lack of willingness of managers and worker owners in parent cooperatives to transform their foreign subsidiaries into worker cooperatives, which contradicts basic cooperative principles like open admission to the cooperative, democratic organization, sovereignty of labor, and the subordinate character of capital (see Heras-Saizarbitoria, 2014). The increasing importance that (capitalist) market economy-related business objectives gain over basic cooperative principles (see Blawat, 2014) represents the second indicator. As Cheney (1997, 2001) has demonstrated for the MAPSA cooperative and Taylor (1994) for the LANA agricultural cooperative, the rhetoric of business economics increasingly shaped discourses among managers and/or among non-managing employees. In the latter case, small farmer members who were strongly committed to the cooperative idea but could not afford to pay their share in collective investments, were urged to leave the cooperative. The MAPSA cooperative—a maker of aluminum wheels that was converted from a traditional capitalist firm to a cooperative in 1991 and 1992 suffered from the introduction of semi-autonomous group work that failed because neo-Tayloristic HRM tools like Total Quality Management (TQM) allowed the employees only low participation opportunities in decision-making (Cheney, 1997, 2001). In an extensive discourse-analytical investigation of business documents from 70 MCC cooperatives, Heras-Saizarbitoria and Basterretxea (2016) demonstrated that the prevailing topics represented conventional business management concepts. While costumer focus/TQM, business excellence, innovation capabilities, policy of internationalization, and strengths of integration into a corporate structure dominated in the majority of cases, only about a quarter of the cooperatives emphasized topics like cooperative values or corporate social responsibility. Thus, discourses in the majority of worker cooperatives seemed to be disconnected from the cooperative principles represented by the MCC. Furthermore, the discrepancy between proclaimed cooperative principles and the experience of those principles in the daily work life reflects a third indication of goal/cultural degeneration, as demonstrated by Heras-Saizarbitoria (2014) in his study of 27 worker-members from 11 MCC member cooperatives. The majority of those workers did not experience the core principles of democratic organization, participatory management, and cooperative education as well integrated in their everyday work (cf. Bretos and Errasti, 2017, 2018). However, the core principle of secure membership and employment was appreciated and applicable to interviewees, forming a starting point for goal/cultural regeneration (cf. the studies on the Eroski group by Flecha and Ngai, 2014; Basterretxea and Storey, 2018).

#### Degeneration tendency and regeneration

This review identified three cases of persistent interweaving of degenerative and regenerative processes (as conceptualized by Storey et al., 2014; Bretos et al., 2020), represented by the Eroski group, the Fagor Ederlan group, and the Copreci cooperative. The Eroski group (founded in 1969) represents the largest MCC enterprise with 30,903 employees in 2018 (Basterretxea et al., 2022). Eroski is a combined consumer and worker cooperative allowing consumer-members and worker-members equal representation in elected governing bodies, namely the general assembly and the Governing Council, elected by all cooperative members. Empirical evidence indicates that the Eroski group has undergone a process of *constitutional degeneration* after incorporation: among its employees the rate of working members (i.e., employee-owners participating equally in ownership and democratic decision-making) fell from about 47% in 2005 to about 33% in 2014 (see, also in the following, Storey et al., 2014; Basterretxea and Storey, 2018). It follows, that two thirds of employees, among them the great majority of those working in hierarchically structured subsidiaries acquired by the Eroski parent cooperative, did not possess democratic decision-making rights at the time of investigation. In contrast, a two third majority of working members within the parent company Eroski S. Coop participated in governing the whole Eroski group. Thus, the biggest enterprise within MCC exhibits strong structural polarization between the parent cooperative and the majority of subsidiaries.

Nonetheless, empirical evidence indicates that several *goal/cultural regeneration* efforts occurred. Following the social expansion strategy adopted by the Mondragon congress in 2003, within the democratic entities in the Eroski group and, similarly, in the Fagor Electrodomésticos group, the Fagor Ederlan group, the Maier group (and, presumably, further MCC enterprise groups that have not been investigated), a debate on the regeneration of democratic principles and social objectives began. It caused the Eroski parent cooperative to transform several of their domestic subsidiaries into a variant of *mixed cooperatives* as an intermediate stage to later integrate them fully into the Eroski parent cooperative (Flecha and Ngai, 2014; Storey et al., 2014). Further participative practices (see, also in the following, Flecha and Ngai, 2014; Storey et al., 2014; Basterretxea and Storey, 2018) were applied in other Eroski subsidiaries not earmarked for cooperativization (i.e., for a transformation into a mixed cooperative): Employees of Eroski stores were allowed to participate in short-term operational or some tactical decisions and in minor capital stakes of the parent cooperative. In 2011, 33.6% of the total employees of the Eroski group had such partial ownership stakes in subsidiaries. Moreover, management and democratic representatives participated in discourses within the Eroski groups and in the MCC self-reflection debate emphasizing the regeneration of democracy and cooperative values. Additionally, an expansion of cooperative education was initiated and employee representatives successfully prevented the modification of egalitarian principles (e.g., concerning profit sharing).

In contrast to Fagor Electrodomésticos, after symptoms of constitutional and goal degeneration had appeared in the course of internationalization caused by global market pressures toward high productivity and quality, the Fagor Ederlan group, an automotive supplier founded in 1963; about 3,600 workers at 16 plants in Spain, China, Brazil, and Slovakia in 2014 (Bretos et al., 2019), succeeded both economically and in the cooperativization of several subsidiaries, such that working members again constituted the majority of employees (65% in 2014, see Bretos and Errasti, 2017; 72% in 2021, see https://www.fagorederlan.com/en/about-us, retrieved April 21, 2022). Thus, the constitutional degeneration tendency was reduced significantly. For example, in the governing board of Ederlan's mixed cooperatives, working members from the subsidiary have a greater representation than the proportion that they would have been allocated on the basis of their part of ownership (the parent cooperative holds the majority of the equity capital) and important decisions require a two-thirds majority. Further, regeneration measures comprised cooperative education parallel to technical training, fostering equality through a policy of job security, reduction of wage gap, horizontal communication, and an employee suggestion system in agreement with the unions. Additionally, participation practices included efforts to humanize production work through job enlargement and enrichment and a reinforcement of participation through forming a delegate committee of the worker members of the governing board (Bretos and Errasti, 2017; Bretos et al., 2018).

Notwithstanding these practices, both Fagor groups showed a structural polarization between the Basque parent cooperatives and several domestic mixed cooperatives, on the one hand, and the foreign subsidiaries that enjoy no democratic and collective ownership rights, on the other hand. Several studies have demonstrated that participative practices and components of HRM were distributed unequally among the foreign subsidiaries of both Fagor groups (Errasti et al., 2016; Bretos and Errasti, 2018; Bretos et al., 2018, 2019). Lean production and TQM seem to be widespread in all subsidiaries, allowing many employees only limited participation opportunities in decision-making. Nonetheless, the studies mentioned above indicate that the subsidiaries differed strongly with regard to job security, professional and cooperative training, internal promotion, pay equity, willingness to cooperate between MCC management and foreign trade unions, and employees' stress at work. A lack of trust between parent coops and foreign subsidiaries is mirrored in the lack of a collectivistic culture and cooperative tradition and a weak initial commitment of workers in the majority of foreign subsidiaries. These findings are mirrored by Errasti's (2015) investigation of 11 Mondragon subsidiaries in the Kunshan Industrial Park in China, However, Errasti (2015) conceded that a more participative and considerate leadership style prevailed there, compared to the authoritarian style normally found in capitalistic Chinese subsidiaries. The inclusion of trade union representatives on the subsidiary's board of directors of the Polish subsidiary Fagor Mastercook (then belonging to the Electrodomésticos group) was a further positive exception (Errasti et al., 2016; Bretos et al., 2019). In contrast to the Catholic social doctrine and humanism of the founders, several studies indicate that not only the subsidiaries but also production plants belonging to the MCC parent cooperatives were affected by semi-Tayloristic principles of work organization, modulated by management-controlled participative practices stemming from lean production and TQM (Cheney, 2001; Basterretxea et al., 2019; Bretos et al., 2019, 2020). This corresponds with findings of an interview study by Heras-Saizarbitoria (2014), in which the majority of 27 worker-owners in 11 MCC cooperatives perceived few possibilities to participate in everyday decision-making (however, the sample was small and not representative for those large enterprises).

In contrast, influenced by the approach of sociotechnical work design, some MCC cooperatives like Fagor Edelan and Copreci have adopted semi-autonomous group work and similar interventions oriented toward the humanization of work (see Taylor, 1994; Cheney, 2001; Bretos and Errasti, 2017; Bretos et al., 2019). Taylor's (1994) study describes the development of competing rhetorical strategies between economic and social goals in the Coprecci cooperative which nurtured a social discourse emphasizing democracy, social transformation, and community development as constituents of goal and cultural regeneration. A more participative reorganization of social councils and the constitution of permanent and *ad hoc* committees and study teams represent further practices to reinforce employees' participation in organizational decision-making and, thus, support constitutional and organizational regeneration of the Coprecci cooperative.

#### Retention

The ULMA group (a supplier and service provider for various industries) demonstrated goal/cultural and constitutional retention through practices (see Table 8) like cell-division as a consequence of identification with the local community: Because of its strong commitment to the Mondragon founding values, the ULMA group consisting of five worker cooperatives encompassing 1,200 employee-owners at the time had left MCC in 1992 after debating threats that the internationalization strategy posed to democratic functioning and community development (Cheney, 1997, 2001). The members of the ULMA group decided to retain their regional grouping instead of adopting the new sectoral reorganization of MCC. This indicates their considerable orientation to promote community-based goals in the region and their strong commitment to the cooperative idea. Eventually, the group re-united with the industrial division of MCC and is represented by one member in the governing council of MCC in 2020 (see https://www.mondragon-corporation.com/urtekotxostena/organos.php?l=en, retrieved April 19, 2022). About 20 years later, 93% of its employees were still cooperative members (Blawat, 2014). This is also due to the fact that the parent cooperative's worker owners are motivated to retain the cooperative identity also in the acquired subsidiaries through practices like cooperativization, reinforcing participation through self-managed group work and intensive sharing of knowledge and information (Flecha and Ngai, 2014).

**Table 8:** Frequencies of conditions, practices, and psychological phenomena of *all forms of retention or regeneration* mentioned (*n* = 6 cases) of the Mondragon Cooperative Corporation.

**Conditions, practices, and psychological phenomena**	**Frequency**	**Enterprise groups and publications**
**1. Organizational/external conditions:**	**1**	
Support through the union	1	Fagor Ederlan group (Bretos et al., 2018^*^)
**2. Democratic or participative practices:**	**24**	
**2.1 Integration and delimitation (boundary management)**				
Linking with broader social movements to promote community-based goals/intercooperative solidarity	1	Grupo ULMA (Cheney, 1997, 2001)
Cooperativization of (capitalist) subsidiaries/remutualization	3	Eroski group (Flecha and Ngai, 2014; Storey et al., 2014^*^); Fagor Ederlan group (Bretos and Errasti, 2017^*^); Maier group (Flecha and Ngai, 2014)
Effort to stay small/resist to grow/cell division	1	Grupo ULMA (Cheney, 1997, 2001)
Support through external expertise	1	EB cooperative (Narvaiza et al., 2017)
**2.2 Value-based human resources management**		
Value-based personnel selection of the CEO	1	EB cooperative (Narvaiza et al., 2017)
Cooperative education and training	3	Eroski group (Flecha and Ngai, 2014); Fagor Ederlan group (Bretos and Errasti, 2017^*^; Bretos et al., 2019^*^); Maier group (Flecha and Ngai, 2014)
Knowledge/information/experience sharing	2	Maier group (Flecha and Ngai, 2014); ULMA group (Flecha and Ngai, 2014)
Job rotation/job enlargement/job enrichment	1	Fagor Ederlan group (Bretos and Errasti, 2017^*^)
**2.3 Support of cooperative culture and climate**		
Open criticism and discussion, permanent requirement for accountability and overt critique of managers	1	Eroski group (Storey et al., 2014^*^)
Social discourses emphasizing democracy, social transformation, and community development	3	Coprecci cooperative (Taylor, 1994)^*^; Eroski group (Storey et al., 2014^*^); Grupo ULMA (Cheney, 1997, 2001, 2004)
Fostering equality	2	Eroski group (Basterretxea and Storey, 2018^*^); Fagor Ederlan group (Bretos et al., 2018^*^)
**2.4 Support of participation**	
Organization of labor: reinforcement of broad-based participation	5	Coprecci cooperative (Taylor, 1994)^*^; Eroski group (Storey et al., 2014^*^); Fagor Ederlan group (Bretos and Errasti, 2017^*^); Maier group (Flecha and Ngai, 2014); ULMA group (Flecha and Ngai, 2014)
**3. Psychological phenomena:**	**5**	
Strong commitment to cooperative idea and to the enterprise/loyalty/“family”/strong willingness to participate	4	EB cooperative (Narvaiza et al., 2017); Eroski group (Basterretxea and Storey, 2018^*^); Grupo ULMA (Cheney, 1997, 2001, 2004; Flecha and Ngai, 2014)
Sacrifices for the organizational community	1	Eroski group (Basterretxea and Storey, 2018^*^)

Several studies described the same conditions, practices, and psychological phenomena for the same enterprise. These phenomena were counted only once for each enterprise. Therefore, the number of publications may exceed the number of counted conditions, practices, and psychological phenomena.

* Degenerative tendency and regeneration activity appear simultaneously in this case.

#### Regeneration

Finally, two cases demonstrate strong tendencies of *goal/cultural regeneration*. The Maier group, a manufacturer of plastic parts and complex injection moldings for the automotive industry (meanwhile encompassing about 3,200 employees, see https://www.maier.es/), started extensive activities of constitutional regeneration through transforming a big subsidiary into a mixed cooperative (Flecha and Ngai, 2014). Further participative practices have been applied to counter degeneration tendencies in the parent cooperative and subsidiaries (see Table 8). These include widespread education in cooperative values and technical-organizational training, measures to increase transparency and knowledge-sharing across all organizational levels, and to reduce wage gaps to foster income equality. Further, principles of sociotechnical work design like job rotation, job enrichment, and semi-autonomous group work as well as employee suggestions systems were introduced at some subsidiaries to transform the Tayloristic work organization. The EB cooperative (anonymized), a manufacturer of copper wires with 170 employees (90% of them are employee-owners) engaged external consultants, psychologists and senior managers with expertise in cooperativism to strengthen the cooperative spirit of newcomers (Narvaiza et al., 2017). These practices helped to recover the cooperative spirit among the members of the cooperative.

Whereas, one of two further studies indicated goal/cultural retention concerning basic values like autonomy, empowerment, egalitarianism, and fairness (Hoffman and Shipper, 2018), the other study showed more ambivalent findings regarding tensions between retention and degeneration of values (Blawat, 2014). However, because both do not refer to specific MCC cooperative groups or cooperatives, but to the overall MCC network, and because the samples of interviewed employees are extremely small, these studies were not included in the table of results.

## Discussion

### Summary of main findings

Summing up the results of research questions 1–3, the present systematic review provides compelling evidence that the majority of investigated democratically structured enterprises operate at least sufficiently economically successful in capitalist market environments, thereby resisting Webb and Webb's (1914) deterministic degeneration thesis and defying the so-called “iron law of oligarchy” (Michels, 1915).

The most frequently reported *organizational/external condition* for the *retention* of organizational democracy was small size of the enterprise. The most widely mentioned *democratic or participative practices* and *psychological phenomena* that foster retention were similar to those of *regeneration* or *retention* as previously identified by several other scholars (e.g., Rothschild-Whitt, 1976; Cornforth, 1995; Bretos et al., 2020). *Participative practices* included activities of boundary management (Ulich, 2011) like linking with broader social movements to promote community-based goals, measures of HRM based on cooperative values like personnel selection, common recruitment and induction procedures as well as cooperative education and training and supporting the further socialization process, knowledge/information/experience sharing and job rotation/job enlargement/job enrichment. Our systematic review revealed measures to support cooperative culture and climate like open criticism and discussion, permanent requirement for accountability and overt critique of managers, social discourses emphasizing democracy, social transformation, and community development and, finally, practices to support employee participation also in operational and tactical decisions in the daily work through a democratic organization of labor as frequently used participative practices. The *psychological phenomena* reported in the analyzed studies encompassed strong commitment to the cooperative idea and to the enterprise/loyalty/framing as “family” and psychological ownership. However, we found some additional practices that seem to foster the retention or regeneration of democratic enterprises that were not reported in earlier conceptual reviews: Fostering equality and the effort of enterprises to stay small and resist growth. The psychological phenomena of employees' mutual perspective taking and caring as well as making sacrifices for the community, and, social inclusion orientation and employees' entrepreneurial motives were also not discussed in earlier reviews.

Overall, the number of enterprises that *fully degenerated* or that showed *forms of degeneration tendencies* was smaller than the number of enterprises categorized as retention or regeneration (36.5 vs. 63.5%). However, the studies included in the systematic review considered *organizational/external conditions* far more relevant for enterprises that have degenerated fully or tendentially than for enterprises categorized as retention or regeneration. The most common conditions reported were market demands, pressure, or strong competition, the (large) size of company and expansion, and the passivity of unions in ownership matters. We found several *non-democratic or non-participative practices* and *psychological phenomena* that had been identified in earlier conceptual reviews as counterproductive to retention, such as excluding new employees from ownership, lack of cooperative education and training, semi-/Tayloristic organization of labor and low participation opportunities in decision-making, and a weak initial commitment to employee ownership. Furthermore, we also detected new practices, which had not been previously mentioned. Two of them represent a deficient cooperative culture or climate, namely fostering inequality, and failing to shape a culture of involvement and deliberation or unfamiliarity with the employee ownership concept and, the third one means that goals of employee ownership were not democratizing, but to gain dividends and secure employment.

The findings concerning MCC (RQ 4) indicate that vast organizational size and integration of an enterprise into the global capitalist market economy do *not automatically determine* organizational democracy to fail. However, permanent tensions between market-induced pressures to adopt principles of capitalistic accounting and values of cooperativism and economic democracy exist. Those tensions can culminate in processes of organizational change that represent simultaneous or successive degenerative and regenerative tendencies (cf. Cheney et al., 2014; Storey et al., 2014; Bretos et al., 2020). The results of this systematic review suggest, that, in the long term and depending on their specific resources, participative practices, and strategic decisions, big democratic enterprises are able to maintain or regenerate democratic and cooperative principles of decision making on a broad scale. Alternatively, they may be able to restore democratic structures only on a smaller scale compared to their earlier life-cycle phases or regenerate by partitioning into separate divisions or enterprises (as in the case of ULMA, see Cheney, 1997, 2001). In less resilient cases, they either fully degenerate and give up democratic principles and collective ownership or stay democratic but fail economically. All those different cases are represented in MCC. Participative practices in MCC subsidiaries are mostly restricted to voice and problem solving with regard to operational—but not tactical or strategic—planning and decision-making. Such conditional participative practices are dominated and channeled by economistic management concepts like semi-Tayloristic lean production and TQM. These were introduced to adapt workers' behaviors to customer demands on the global markets for home appliances, industrial facilities, or commercial vehicles. Depending on circumstances, such restricted participation may support employees in developing basic social competences and organizing skills that are expedient for projects of democratic regeneration. However, they cannot replace cooperativization or, at least, projects of “democratic Taylorism”, which include strategic co-determination by representatives of the workers (see Adler, 1995).

Included studies on the John Lewis Partnership (JLP) in the UK corroborate our rejection of the deterministic degeneration hypothesis. In contrast to the MCC network, JLP represents a single retail enterprise group. Nearly all of the 86,700 employees have the right to participate in strategic decision-making *via* their elected representatives in the JLP council (encompassing representatives of all department stores) and the JLP management board, and in collective ownership *via* a trust controlled by democratic representatives (Hoffman and Shipper, 2018). While both main boards of representative democracy on the level of the enterprise group continued to exist since several decades, the study by Cathcart (2013, 2014) indicated a moderate organizational degeneration tendency because democratic branch councils were replaced with merely consultative branch forums; the same happened in 29 from 37 department stores. However, parallel to this limitation of democracy, an ongoing debate on the importance of democratic, social and economic goals evolved, whereby economistic management positions did not gain dominance over the cooperative values of the JLP constitution. Further, several democratic renewal initiatives have been developed indicating a case of goal/cultural retention at the time of the study (see also Storey et al., 2014; Storey and Salaman, 2017; Hoffman and Shipper, 2018). Thus, neither overly optimistic assessments (e.g., Forcadell, 2005) nor totally pessimistic views (like Kasmir, 2016) draw a realistic picture of the democratic situation in the MCC or the JLP.

### Theoretical and practical implications

**Figure 2:**
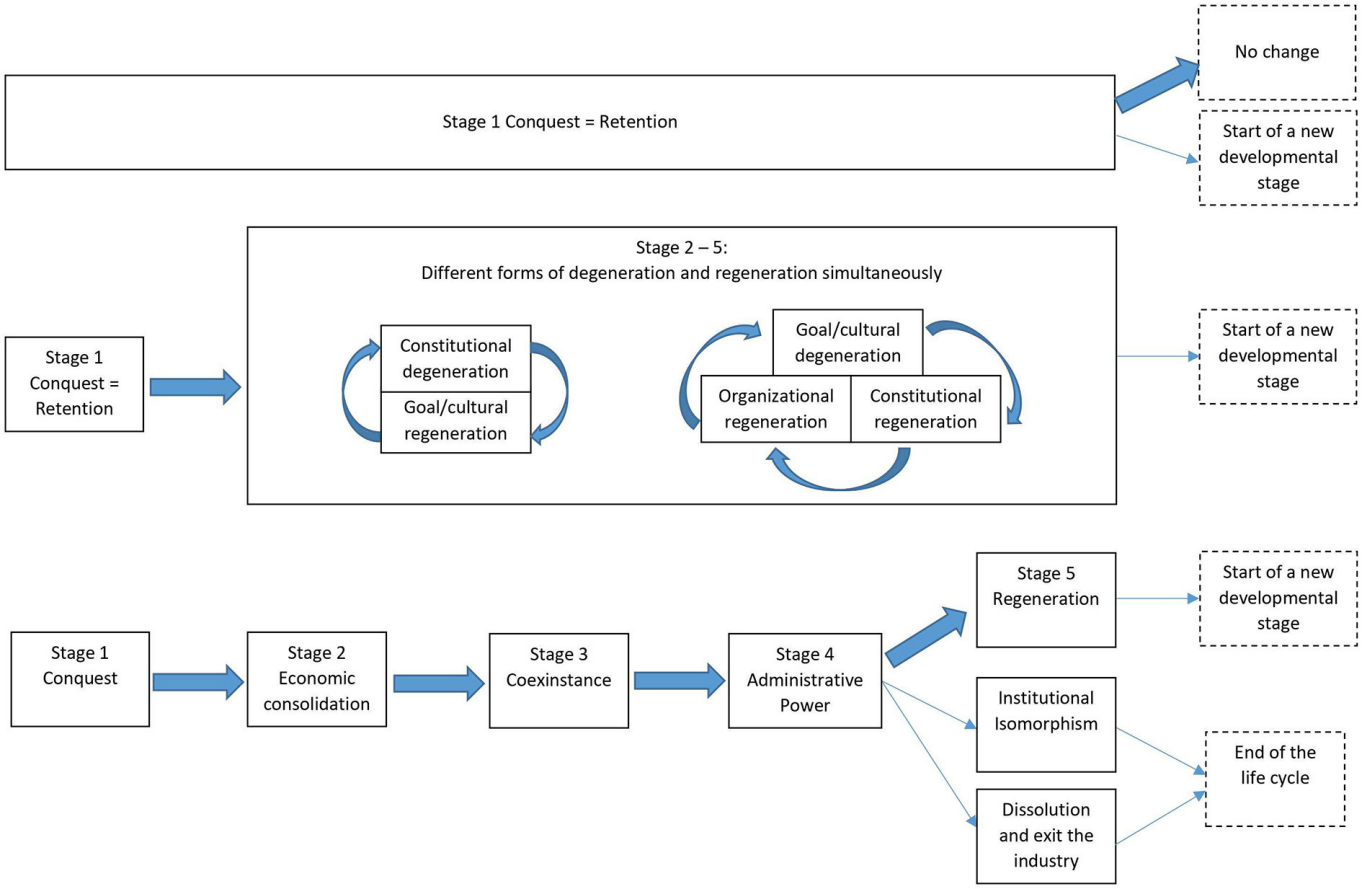
Stages of the democratic enterprises' life cycle (extension of Bretos et al., 2020's life cycle model).

Theoretically, we see the most important implication of this systematic review in the further development of proposed life-cycle models of cooperatives (Batstone, 1983; Meister, 1984; Bretos et al., 2020) based on empirical evidence. The extended model is substantiated with the above summarized concrete organizational/external conditions, democratic/participative practices and psychological phenomena, which are associated with different stages of a democratic enterprise' life-cycle. Figure 2 provides an overview of this revised model. The lower part of the model reflects Bretos et al.'s (2020) five stages of cooperative life-cycles, which, until now, has been the most comprehensive and best developed model. Stages 1 (conquest) to 4 (administrative power) mirror Meister's (1984) life-cycle model of degeneration. Stage 5 allows for processes of regeneration, but also includes pathways of institutional isomorphism (capitalistic solidification) or dissolution and exit from the industry. After regeneration, a new life-cycle may start, however, after institutional isomorphism and dissolution and exit the industry, the life cycle of a cooperative will end (Bretos et al., 2020). In our systematic review we identified enterprises in all of the proposed stages of Bretos et al.'s (2020) model. However, we designated Stage 1 as *retention* instead of conquest, Stage 2 as *moderate goal/cultural degeneration tendency* instead of economic consolidation, Stage 3 as *organizational and constitutional degeneration tendency* instead of coexistence, Stage 4 as *full degeneration* instead of administrative power, and, finally, Stage 5 was maintained as a possible phase of *regeneration*.

Additionally, and neither included in the initial model (Meister, 1984) nor in the revised life-cycle models (Batstone, 1983; Bretos et al., 2020), we found that the majority of investigated democratic enterprises resisted degeneration and degeneration tendencies. The majority of enterprises (half of them small-, half of them medium-sized) categorized as *retention* (upper part of Figure 2), remained in Stage 1 for long periods of time (about two thirds of them at least 20 years) and did not underwent democratic changes. Such processes were also proposed by Rothschild-Whitt (1976), however they were never included in a cooperative's life-cycle model. The Cheese Board Collective in Berkeley, USA (Gupta, 2014) is a concrete example for such a retention process. This small grass-roots worker-cooperative maintains direct democracy through frequent general assemblies, a comprehensive job rotation scheme, and founding spin-offs (instead of organizational growth) without creating representative democratic boards or hiring non-participating or non-owning employees. Finally, it may also happen that some enterprises, which remained in Stage 1 for a long period of time, will may experience unfavorable conditions in future. As a consequence, they will drive the adoption of non-democratic practices or psychological phenomena that force them into a new developmental stage in the sense of Bretos et al.'s (2020) life-cycle model.

In the middle part of Figure 2, we depict two further possible configurations for which we found evidence. In MCC, two large cooperatives showed forms of constitutional degeneration tendency and goal/cultural regeneration simultaneously. Furthermore, one cooperative was facing a goal/cultural degeneration tendency while simultaneously undergoing constitutional and organizational regeneration. Theoretically, other combinations of different forms of re- and degeneration may occur at the same time. Such simultaneous processes may result under specific conditions at the outset of a new developmental stage. Empirically, these simultaneous configurations are not fully in line with the linear structure of Bretos et al.'s (2020) stage model (especially the path from Stage 2–5). We suggest that there are several pathways for democratic enterprises to develop simultaneously and in different causal sequences.

Practical implications can be derived from the identified practices that foster the retention of democratic enterprises, providing helpful guidelines for their continued existence. Furthermore, described retention and regeneration practices may be fruitful measures for democratizing hierarchically structured enterprises. Findings of this review study suggest that the larger a democratic enterprise grows the more necessary it will become to integrate the big majority (or all) of the employees directly into practices of decision-making. Otherwise, democratic structures run the risk of becoming eroded, that is, there will be a threat of degeneration processes. This does not mean that large democratic corporations must necessarily be split up (although in some cases this would be better for the inter-/national economy). Rather, several of the reviewed case studies indicate that structures of representative democracy can be combined with practices of direct democratic decision-making on the level of the department or the work group and, particular participatory practices even on the level of the organization. For example, as the findings about MCC (see Section Research question 4: The Mondragon Cooperative Cooperation Network and its forms of transformation as well as relating conditions, practices, and psychological phenomena) converge with studies on more conventional enterprises, semi-autonomous work groups can be established in industrial areas characterized by small or medium series production or, in form of self-managed teamwork, in specific domains of service or administrative work (see a review by Ulich, 2011; Lee and Edmondson, 2017). Here the members of the working groups in a respective company are participating directly in collective tactical or operational decisions associated with planning, coordinating, and controlling the carrying out of the necessary, interrelated subtasks that serve the production of jointly produced products or services. Direct democratic group work lets employees experience a sense of control and mastery and counteracts the risk of feeling alienated from large enterprises practicing representative democracy in which only the elected organization members can directly play a decisive part (Bartoelke et al., 1982).

In cases where democratization of certain work activities does not seem feasible, some of the case studies reviewed demonstrate other ways in which a considerable number of employees can be directly involved. In democratic enterprises like Cooperative Home Care Associates (Berry and Schneider, 2011), JEBA Manufacturing and Supply Inc. (Boguslaw and Taghvai-Soroui, 2018), in several John Lewis department stores (Nicholson et al., 2020), or in the MCC cooperative Coprecci (Taylor, 1994) several organization members were delegated to working groups, committees or project groups in which certain strategic or tactical decisions were prepared for general meetings of those enterprises. These include economic, technical (including product or process quality), and personnel-related (including occupational health and safety) issues or decisions on caregiving or support of the local communities.

Provided that these delegates are exchanged after longer periods of function and, thus, numerous employees gradually will carry out responsible planning tasks or problem-solving tasks together, this democratic practice also represents an important method of counteracting degenerative tendencies.

Only a few of the reviewed studies included or suggested any method or intervention to prevent organizational or goal degeneration. Mainly, these referred to larger enterprises that were governed based on representative democracy. In such cases, frequent direct communication on substantial decisions (including different options) between employees eligible to vote and their representatives, is recommendable. Based on their contextual knowledge, the voters can then point out problems or alternatives concerning current planning of the representative body. In medium-sized companies, this participative communication may be realized spontaneously as needed if this doesn't disturb production flows (see Greenberg, 1984; Atzeni and Ghigliani, 2007). In large enterprises, recurring meetings between representatives and their organizational units should be institutionalized, e.g., monthly or every 2 months.

Notwithstanding this, it is surprising that in hardly any of the reviewed cases established participatory intervention methods from organizational development were used. After all, these participatory methods, following the work of Kurt Lewin and his successors, as well as the sociotechnical systems approach, are at the starting point of a democracy-oriented research and design of democratic work systems (see Trist and Murray, 1993). Democratic Dialogue conference (Gustavsen, 1992), Search Conference (Emery and Purser, 1996), Future Search (Weisbord and Janoff, 2000), Twenty-first Century Town Meeting (Lukensmeyer and Brigham, 2002), or Constructive Controversy (Johnson et al., 2006) are all prominent examples. These conferences, workshops, or encounters enable employees to directly participate democratically in planning or design processes in addition to electing their representatives. These approaches offer a wide range of possibilities involving a large number of organization members in annual—or more frequent—retreats (all together or specific groups at different times) in joint planning of the further development of the company or specific departments. Various methods are used for participative preparation of product and process innovation, others for resolving factual conflicts in development processes. Furthermore, these large-group methods, comprising exchanges between parallel work in small groups and integration of the proposals in plenums, also aim to promote democratic, social, communicative, or cognitive-moral learning processes among the participants. We encourage future studies to investigate how these intervention methods reinforcing broad-based participation in democratic enterprises will interact with further participative practices in terms of value-based HRM and support of cooperative culture and climate, like those that our review has identified. Against the background of our findings, it can be assumed that such participatory interventions together with participative practices in everyday work may facilitate participants' commitment to the cooperative idea and to their enterprise, their collective psychological ownership, their prosocial behavior, and their readiness to make sacrifices for their company.

Thus, the findings of this review study may contribute knowledge to the multifarious governmental programs, interventions and political activities aiming all over the world at a democratic and ecological transformation of the economy in the global context that, for example, Ferreras et al. (2022) have proposed. Thus, extracted results are of high practical applicability.

### Limitations and future research

A first major limitation concerns the available data for the described enterprises. The analytical depth and level of detail of included qualitative studies varied considerably. Some studies describe in great detail the structures, bodies, principles and typical practices of organizational democracy with regard to the three forms of transformation. In other studies, democratic structures and participatory practices were outlined only sparsely and vaguely. Surprisingly, the included studies only rarely provided a detailed and comprehensive analysis of (a) what percentage of those working in a given democratic enterprise participated, (b) how frequently, (c) in which specific strategic or tactical decisions, (d) directly or *via* elected representatives, (e) through what panels or practices, (f) or how the employees participated in the equity capital of their company. Frequently, some of this information was missing. The inclusion of larger parts of staff members from different departments and hierarchical levels through standardized survey questionnaires (see, e.g., IDE International Research Group, 1981; Heller et al., 1988; Weber and Unterrainer, 2012) would have allowed to assess the constitutional and organizational degenerative or regenerative tendencies with higher internal validity than this was typically done in the studies reviewed here. In future research, such standardized questionnaires assessing the above aspects should be integrated into mixed methods designs wherever possible.

A second limitation refers to the theoretical foundation of the identified psychological phenomena. Focusing on organizational change and sustainability, organizational psychology has suspiciously neglected research on organizational democracy, which was also demonstrated in our meta-analysis (Weber et al., 2020). Therefore, it is not surprising that, although the majority of included studies pursue psychologically relevant questions, discovered phenomena are often only superficially (if not at all) related to established theories, constructs, or models of work, organizational, or social psychology. This shortcoming should be addressed in future studies using the findings of this systematic review to collaborate across disciplines in both theory and hypothesis development and in planning mixed-methods research designs. If the—politically induced—artificial disciplinary divisions were abandoned in favor of a scientific cross-fertilization of perspectives, this would offer opportunities to better understand the interactions among psychological, organizational, economic, and social characteristics and processes. This would contribute to shaping the frequently invoked social and ecological transformation locally as well as globally.

Furthermore, eight enterprises that were categorized as retention provided only cross-sectional information. This means that at the time of investigation all indicators pointed to a retention. Since information was missing on the enterprise' development from its founding until the time of study, it is possible that these enterprises earlier went through a cycle of degeneration and were at the time of investigation already fully regenerated. However, since the democratic practices and psychological phenomena of retention and regeneration processes turned out to be very similar, this limitation seems to be less of a concern.

Another limitation refers to the respective samples of interviewees. Although in several cases, some theoretical sampling procedures were applied, especially studies on big enterprise groups like the MCC network or John Lewis Partnership cannot claim representativeness.

Included research designs are only limitedly comparable. Qualitative methodology is much more diverse and less standardized than quantitative questionnaire surveys. Specifically, the latter facilitate systematic reviews on highly aggregated characteristics. However, this means that possible external and internal organizational influencing factors and complex interaction processes, which are due to the specific context of concrete cases, are overlooked.

A final limitation applies only to MCC. The nine cases that met the inclusion criteria were representing only a part of the MCC enterprises. In this sub-sample, big cooperative groups were overrepresented compared to small or medium-sized MCC cooperatives.

## Conclusion

The present article reviews qualitative research on democratically structured enterprises, published between 1970 and 2020, with respect to their potential for retention, degeneration, and regeneration. We were able to provide strong evidence that the pessimistic view regarding the short-lived survival of democratic enterprise in a capitalistic market environment—the degeneration thesis by Webb and Webb (1914) and further scholars—is completely overblown. Out of 83 investigated enterprises 50 showed no signs of degeneration or even degeneration tendencies, and most of the enterprises that showed degeneration tendencies were still far away from full degeneration [e.g., Equal Exchange (USA), Cooperative Home Care Associates (USA), One World Natural Grocery (USA), or Opel Hoppmann (Germany)]. The present study therefore raises the hope that democratically structured enterprises in the sense of “real utopias” (Wright, 2010) have the potential of contributing to a socio-ecological transformation especially by connecting and networking with broader social movements to promote a more socially and ecologically just and sustainable economy and humanist society.

## Data availability statement

The original contributions presented in the study are included in the article/Supplementary material, further inquiries can be directed to the corresponding author.

## Author contributions

All authors listed have made a substantial, direct, and intellectual contribution to the work, and approved it for publication.

## Funding

This article was supported by the Publishing Fund of the University of Innsbruck in cooperation with the Faculty of Psychology and Sport Science. The funders had no role in study design, data collection and analysis, decision to publish, or preparation of the manuscript.

## Conflict of interest

The authors declare that the research was conducted in the absence of any commercial or financial relationships that could be construed as a potential conflict of interest.

## Publisher's note

All claims expressed in this article are solely those of the authors and do not necessarily represent those of their affiliated organizations, or those of the publisher, the editors and the reviewers. Any product that may be evaluated in this article, or claim that may be made by its manufacturer, is not guaranteed or endorsed by the publisher.
