# Supplementary material for: Organizational and psychological features of successful democratic enterprises: A systematic review of qualitative research
Source: Front Psychol. 2022 Nov 4;13:947559. doi: 10.3389/fpsyg.2022.947559 (PMC9672673; doi:10.3389/fpsyg.2022.947559)
Supplement: Supplementary file 1 [file Data_Sheet_1.docx]

**Supplementary Table 1**

**Applied search terms:**

(organi?ational W0 democracy) OR (industrial W0 democracy) OR (workplace W0 democracy) OR (worker* W0 democracy) OR (worker* W0 self-management) OR (employee* W0 self-management) OR (employee* W0 ownership) OR (employee* W0 stock W0 ownership) OR (worker* W0 ownership) OR (worker W0 owned W0 organi?ation*) OR (worker W0 owned W0 firm*) OR (worker W0 owned W0 enterprise*) OR (worker W0 owned W0 corporation*) OR (employee* W0 owned W0 organi?ation*) OR (employee* W0 owned W0 firm*) OR (employee* W0 owned W0 enterprise*) OR (employee* W0 owned W0 corporation*) OR (democratic W0 firm*) OR (democratic W0 compan*) OR (democratic W0 corporation*) OR (democratic W0 organi?ation*) OR (worker* W0 cooperative*) OR (producer* W0 cooperative*) or (cooperative W0 enterprise*) OR (worker* W0 co-operative*) OR (producer* W0 co-operative*) or (co-operative W0 enterprise*) OR (self-governed W0 enterprise*) OR (self-governed W0 firm*) OR (self-governed W0 corporation*) OR (self-governed W0 organi?ation*) OR (labor W0 managed W0 enterprise*) OR (labor W0 managed W0 firm*) OR (labor W0 managed W0 corporation*) OR (labor W0 managed W0 organi?ation*) OR (collectivist W0 organization*) OR (collective W0 organi?ation*) OR (collectivist W0 enterprise*) or (collective W0 enterprise*) OR (collectivist W0 firm*) or (collective W0 firm*) OR (collectivist W0 corporation*) or (collective W0 corporation*) OR (employee* W0 buyout) OR (employee* W0 buy-out) OR (democratic W0 management)

**Supplementary Table 2.**

Democratic enterprises with different forms of full degeneration and degenerating tendencies

| **Enterprise** | **Country** | **Number of employees** | **Founding year / founding democratic model** | **Tenure of democratic practice (years)** | **Proportion of worker owners** | **Corresponding publications** |
| --- | --- | --- | --- | --- | --- | --- |
| **Full Degeneration** | | | | | | |
| **Full Degeneration (more than one form)** | | | | | | |
| 1. Fishery & Restaurant | Scotland | 100 | 1978/2002 | ~10 | 0 % | Sacchetti & Tortia (2020) |
| 1. LC (anonymized) Printing | France (Breton) | 15 | 1975/1975 | 41 | 0% | Narvaiza et al. (2017) |
| 1. Kompro (service firm) | Estonia | 100-200 |  | 9 | Less than 33 % | Kalmi (2002) |
| 1. Phoenix (global sales of cutting machines for large-scale work-pieces) | Germany | 13-175 | 1996-2011 | 16 | 0 % | Kranz & Steger (2016) |
| **Full constitutional Degeneration** | | | | | | |
| 1. Algoma Steel | Canada | 7175 (1992);  3650 (2002) | 1992/1992 | 10 | Less than 33 % | Lindenfeld (2001) |
| 1. Omega Ltd. manufacturing of boilers | Brazil (South Eastern countryside) | ~130 | 2000/2000 cooperative; 2005 converted into limited liability Co. | 9 | Less than 33 % | Bittencourt Meira (2014). |
| **Full goal/cultural Degeneration** | | | | | | |
| 1. Union Taxi Cooperative | USA (Denver) | 262 | 2008/2008, 2015 separation from the union | ~8 | More than 50 % | Borowiak & Ji (2019); Ji (2018) |
| **Degeneration tendency** | | | | | | |
| **Degeneration tendency (more than one form)** | | | | | | |
| 1. Community Recreation Cooperative (Manufacturer of “recreational gear”) | USA | 90 | Late 1970s/Late 1970 | 25 | More than 50 % | Schoening (2006) |
| 1. Cooperativa Insieme | Italy | ~120 | 1970ies | ~40 | More than 50 % | Pansera & Rizzi (2020) |
| 1. SAL6 metal manufacturing | Spain (Basque) | 47 | 1993/1993 | 12 | More than 50 % | Jensen (2011) |
| 1. SAL7 metal manufacturing | Spain (Madrid) | 57 | 1997/1997 | 8 | More than 50 % | Jensen (2011) |
| **Moderate constitutional degeneration tendency** | | | | | | |
| 1. Cooperative Home Care Associates (CHCA) | USA (New York, Bronx) | 1700 | 1985/1985 | 24 | More than 50 % | Berry & Schneider (2011) |
| Cooperative Home Care Assoc. | USA (South Bronx) | 2300 | 1985/1985 | 30 | More than 50 % | Kennelly & Odekon (2016) |
| 1. Equal Exchange | USA | 126 | 1986/1986 | 32 | More than 50 % | Hoffman & Shipper (2018) |
| Equal Exchange (fair trade food / beverages) | USA (West Bridgewater) | 150 | 1986/1986 | 29 | More than 50 % | Kennelly & Odekon (2016) |
| 1. Nir Taxi Station Coop | Israel (Tel Aviv) | 52 | 1931 / 1931 | 66 | More than 50 % | Darr (1999) |
| 1. Printing House (industry) | Estonia | 100-200 | Beginning of 1990 | 9 | More than 50 % | Kalmi (2002) |
| 1. SAL2 metal manufacturing | Spain (Basque) | 58 | 1985/1985 | 20 | More than 50 % | Jensen (2011) |
| 1. SAL4 metal manufacturing | Spain (Basque) | 250 | 1989/1989 | 16 | More than 50 % | Jensen (2011) |
| 1. SAL5 metal manufacturing | Spain (Basque) | 172 | 1991/1991 | 14 | More than 50 % | Jensen (2011) |
| **Strong constitutional degeneration tendency** | | | | | | |
| 1. Atlas Container Corporation; Production and selling of cardboard boxes | USA (Mid-Atlantic) | ~ 200 | ? / 1991 | 21 | Less than 50 % | Calo & Shipper (2018) |
| Atlas Container | USA | 220 | 1968/1991 | 27 | Less than 50 % | Hoffman & Shipper (2018) |
| **Organizational degeneration tendency** | | | | | | |
| 1. Comercio y Justicia (newspaper); worker recuperated enterprises (ERTs) | Argentina (Buenos Aires & Cordoba) | 71 | ? probably from 2001-2004 | ~5-9 | More than 50 % | Vieta (2012) |
| Comercio y Justicia (newspaper); worker recuperated enterprises (ERTs) | Argentina (Buenos Aires & Cordoba) | 71 | ? probably from 2001-2004 | ~5-9 | More than 50 % | Vieta (2014) |
| 1. John Lewis Partnership (48 department stores, 350 Waitrose supermarkets) | UK | 81.000 | 1864 / 1929 | At least 78 | More than 50 % | Cathcart, A. (2013) |
| John Lewis Partnership (48 department stores, 350 Waitrose supermarkets) | UK | 81.000 | 1864 / 1929 | 78 | More than 50 % | Cathcart, A. (2014) |
| John Lewis Partnership | UK | ~90.000 |  | ~86 | More than 50 % | Basterretxea, I., & Storey, J. (2018) |
| John Lewis Partnership | UK | 86700 | 1864/1928 | 90 | More than 50 % | Hoffman & Shipper (2018) |
| John Lewis Partnership | UK | 83000 | 1883/1929 | 90 | More than 50 % | Nicholson et al. (2020) |
| John Lewis Partnership | UK | 91.000 | ?/1930ies | ~80 | More than 50 % | Storey, Basterretxea, & Salaman (2014) |
| John Lewis Partnership | UK | ~90.000 | ? | ? | More than 50 % | Storey & Salaman (2017) |
| 1. One World Natural Grocery | USA (CA) | Ca. 250 | mid-1970s/ mid-1970s | More than 30 | More than 50 % | Meyers (2011) |
| One World Natural Grocery | USA (CA) | Ca. 250 | mid-1970s/ mid-1970s |  | More than 50 % | Meyers & Vallas (2016) |
| One World Natural Grocery | USA (CA) | Ca. 250 | mid-1970s/ mid-1970s | More than 30 | More than 50 % | Meyers (2006) |
| 1. Opel Hoppman Siegen | Germany | 200 | 1937/1974 | 34 | More than 50 % | Jochmann-Döll & Wächter (2008) |
| 1. People’s Daily Bread Bakery | USA (CA) | Ca. 100 | mid-1970s/ mid-1970s | More than 30 | More than 50 % | Meyers & Vallas (2016) |
| People’s Daily Bread Bakery | USA (CA) | Ca. 100 | mid-1970s/ mid-1970s | More than 30 | More than 50 % | Meyers (2011) |
| **Goal/Cultural degeneration tendency** | | | | | | |
| 1. Estre (industry) | Estonia | 100-200 | Beginning of 1990 | 9 | More than 50 % | Kalmi (2002) |
| 1. Puurmani (agriculture) | Estonia | Ca. 300 |  | 9 | More than 50 % | Kalmi (2002) |
| 1. SAL1 metal manufacturing | Spain (Basque) | 23 | 1982/1982 | 23 | More than 50 % | Jensen (2011) |

**Supplementary Table 3.**

Democratic enterprises that resisted the degeneration (Retention)

| **Enterprise** | **Country** | **Number of employees** | **Founding year / founding democratic model** | **Tenure of democratic practice (years)** | **Proportion of worker owners** | **Corresponding publications** |
| --- | --- | --- | --- | --- | --- | --- |
| **Retention** | | | | | | |
| **Cross-sectional information** | | | | | | |
| 1. ABC Distribution (industrial supplies distribution firm) | 1 branch in USA, 9 branches in Canada (Ontario) | 285 | 1955/1973 (49% EO) /1995 (100% EO) | 23 | More than 50 % | Harrison, Singh & Frawley (2018) |
| 1. Engineering service | UK (North England) | 32 | ? | ~20 | Probably more than 50 % | Wren (2020) |
| 1. Hermann Miller | USA | 7000 | 1905/1927 | 91 | Less than 50 % | Hoffman & Shipper (2018) |
| 1. Maryland Brush Comp. | USA | < 50 | 1851/1990 | 28 | More than 50 % | Hoffman & Shipper (2018) |
| 1. Nundah Community Enterprise Cooperative (Café / park and garden maintenance) | Australia (Brisbane) | 26 | 1998/1998 | 20 | More than 50 % | Westoby & Shevellar (2019) |
| 1. SRC Holdings | US | 1500 | 1983/1983 | 35 | Less than 50 % | Hoffman & Shipper (2018) |
| 1. Tuckshop co-op (convenience store inside a university campus) | Hongkong | 8 | 2001/2001 | At least 6 | More than 50 % | Ng & Ng (2009) |
| 1. W.L. Gore & Associates | US | 9600 | 1958/1958 | 60 | More than 50 % | Hoffman & Shipper (2018) |
| **Longitudinal information** | | | | | | |
| 1. Artés Gráficas Chilavert (print shop); worker recuperated enterprises (ERTs) | Argentina (cities of Buenos Aires and Cordoba) | 14 | ? probably from 2001-2004 | ~5-9 | More than 50 % | Vieta (2012) |
| Artés Gráficas Chilavert (print shop); worker recuperated enterprises (ERTs) | Argentina (cities of Buenos Aires and Cordoba) | 14 | ? probably from 2001-2004 | ~5-9 | More than 50 % | Vieta (2014) |
| 1. Bicycle hire and sales | UK (Scotland) | 4-12 | Probably from 1977 | 4-5 | More than 50 % | Oliver (1984) |
| Recycles: Bicycle shop & hire; | UK (Scotland) | 7 |  | 6 | More than 50 % | Cornforth (1995) |
| Bicycles repair & retail (Coop) | UK (Scotland) | 170 | 1977/1977 | ~35 | More than 50 % | Sacchetti & Tortia (2020) |
| 1. Canteen co-op (at a university) | China (Hongkong) | 8 | 2002/2002 | At least 5 | More than 50 % | Ng & Ng (2009) |
| 1. Care Store Coop; University shop for foods and grocery | China (Hongkong) | 11 | 2001/2001 | 12 | More than 50 % | Dai et al. (2019) |
| 1. Caring Home Services (CHS) non-for-profit cooperative | USA (rural area) | 30 (2001);  81 (2006) | 1981/2001 | 5 | More than 50 % | Majee & Hoyt (2009) |
| Caring Home Services (CHS) non-for-profit cooperative | USA (rural area) | 81 (2006) | 1981/2001 | 5 | More than 50 % | Majee & Hoyt (2010) |
| 1. Cheeseboard, retail store | USA (Berkeley) | 8 (1971);  22 (1978) | 1967/1971 | 7 | More than 50 % | Jackall (1984) |
| Cheese board collective | USA (Berkeley) | 55 | 1967/1971 | 43 | More than 50 % | Gupta (2014) |
| 1. Cleaning co-op | China (Hongkong) | 18 | 2002/2002 | At least 5 | More than 50 % | Ng & Ng (2009) |
| 1. Clínica Junín (medical clinic); worker recuperated enterprises (ERTs) | Argentina (Buenos Aires & Cordoba) | ~30 | ? probably from 2001-2004 | ~5-9 | More than 50 % | Vieta (2012) |
| Clínica Junín (medical clinic); worker recuperated enterprises (ERTs) | Argentina (Buenos Aires & Cordoba) | ~30 | ? probably from 2001-2004 | ~5-9 | More than 50 % | Vieta (2014) |
| 1. Collective Copies Cooperative (printing services) | USA (Massachusetts) | 13 | 1982/1982 | More than 25 | More than 50 % | Cornwell (2012) |
| 1. Collective Traders (catering service) | UK | 15 | 2010/2010 | 5 | More than 50 % | Langmead (2016) |
| Collective Traders (catering service) | UK | 15 | 2010/2010 | 5 | More than 50 % | Langmead (2017) |
| 1. Coop Cap; Taxi cooperative | USA (Midwest) | ~200 | ? | More than 20 | More than 50 % | Hoffmann (2005) |
| Coop Cap; Taxi cooperative | USA (Midwest) | 150 | ? | More than 20 | More than 50 % | Hoffmann (2016) |
| Coop Cab | USA (Midwest) | 150 | ? | More than 20 | More than 50 % | Hoffmann (2003) |
| Coop Cab | USA (Midwest) | 150 | ? | More than 20 | More than 50 % | Hoffmann (2006) |
| 1. Dome Hotel | North-Cyprus | 30 | ? /2008 | ~8 | Less than 50 % | Timur & Timur (2016) |
| 1. Food wholesale | UK (North England) | 185 | ? | ~30 | More than 50 % | Wren (2020) |
| 1. Food retail | UK (North England) | 54 | ? | ~15 | More than 50 % | Wren (2020) |
| 1. Homecare service (EO) | Scotland | 400 | ?/2004 | ~ 7 | More than 50 % | Sacchetti & Tortia (2020) |
| 1. Hotel BAUEN | Argentina | 130 | 1978/2003 | 12 | More than 50 % | Sobering (2016) |
| Hotel BAUEN | Argentina | 130 | 1978/2003 | 12 | More than 50 % | Sobering (2019) |
| 1. JEBA Manufacturing and Supply Inc. | USA | ~550 | ~1979 / 1986 à 2000; 10-year seller-financed buy-out plan | ~18 | More than 50 % | Boguslaw & Taghvai-Soroui (2018) |
| 1. Lake: Language school period, | UK | 8 | studied 1978–1985 | 7 | More than 50 % | Cornforth (1995) |
| 1. Natura natural foods cooperative | USA | ~40 | 1970ies | More than 40 | More than 50 % | Ashforth & Reingen (2014) |
| 1. Organix Co-op (food distr.) | UK (mid-North of England) | 50 | ? | More than 20 | More than 50 % | Hoffmann (2016) |
| Organix Coop (food distr.) | UK | 50 | ? | More than 20 | More than 50 % | Hoffmann (2006) |
| 1. Plywood cooperative 1 | USA (Pacific-Northwest) | 90 – 169 | ? | More than 20 | More than 50 % | Greenberg (1980) |
| Plywood cooperative 1 | USA (Pacific-Northwest) | 80 – 350 | Some were founded soon after world war II | More than 20 | More than 50 % | Greenberg (1984) |
| 1. Plywood cooperative 2 | USA (Pacific-Northwest) | 90 – 169 | ? | More than 20 | More than 50 % | Greenberg (1980). |
| Plywood cooperative 2 | USA (Pacific-Northwest) | 80 to 350 | Some were founded soon after world war II | More than 20 | More than 50 % | Greenberg (1984); |
| 1. Plywood cooperative 3 | USA (Pacific-Northwest) | 90 – 169 | ? | More than 20 | More than 50 % | Greenberg (1980). |
| 1. SAL3 (100 % members) metal manufacturing | Spain (Madrid) | 22 | 1986/1986 | 19 | More than 50 % | Jensen (2011) |
| 1. Scopex (sheet metal factory) | France | 25 | ? | 30 | More than 50 % | Jaumier (2017) |
| 1. Scott Bader (manufacturing polyesters & polymers) | UK (North of England) | 220 (1960)  400 (1989) | 1921/1951 | 1960: 9;  1989: 38; | More than 50 % | Hadley & Goldsmith (1995) |
| Chemical Cooperative <Scott Bader Commonwealth> | UK (North of England) | 156 | 1921 / 1951 | 52 | More than 50 % | Hoffmann (2016). |
| 1. The Good Co-operative (grocery shop) | Poland | 8 | 2013/2013 | ~6 | More than 50 % | Kociatkiewicz, Kostera, & Parker (2020) |
| 1. The Vegan Place (Catering & Restaurant) | Poland | 11 | 2013/2013 catering; 2015/2015 restaurant | ~6 | More than 50 % | Kociatkiewicz, Kostera, & Parker, M. (2020) |
| 1. Tower Colliery Worker Cooperative | UK (Wales) | 240 | ? / 1993 | ? | More than 50 % | Hoffmann (2001) |
| Tower Colliery Worker Cooperative | UK (Wales) | 239 | ? | 10 | More than 50 % | Hoffmann (2016). |
| Coal Coop | UK (Wales) | 239 | ? | ? | More than 50 % | Hoffmann (2006) |
| 1. Unión Solidaria de Trabajadores (UST) (construction factory) | Argentina (Greater Buenos Aires) | 65 | ? / presumably 2002 | 5 | More than 50 % | Atzeni & Ghigliani (2007) |
| Unión Solidaria de Trabajadores (construction, maintenance) | Argentina (Greater Buenos Aires) | ~90 | 2004 | ~5-9 | More than 50 % | Vieta (2012) |
| Unión Solidaria de Trabajadores (construction, maintenance) | Argentina (Greater Buenos Aires) | ~90 | 2004 | ~5-9 | More than 50 % | Vieta (2014) |
| 1. Vegetal (foods) | UK | ~15 | 1986/1986 | 28 | Probably more than 50 % | Langmead (2016) |
| Vegetal (foods) | UK | ~15 | 1986/1986 | 28 | Probably more than 50 % | Langmead (2017) |

**Supplementary Table 4.**

Democratic enterprises with different forms of regeneration

| **Regeneration** | | | | | | |
| --- | --- | --- | --- | --- | --- | --- |
| **Enterprise** | **Country** | **Number of employees** | **Founding year / founding democratic model** | **Tenure of democratic practice (years)** | **Proportion of worker owners** | **Corresponding publications** |
| **Constitutional regeneration** | | | | | | |
| 1. Pioneer Valley Photovoltaics | USA (Greenfield) | 16 (2008) | 2002/2003 | 13 | More than 50 % | Kennelly & Odekon (2016) |
| **Organizational regeneration** | | | | | | |
| 1. SAMITI (worker cooperative) service: civil maintenance and cleaning | India | 125 | 1992/1992 | ~7 | More than 50 % | Varman & Chakrabarti (2004) |
| 1. Suma Wholefood: Wholesaling | UK | 35 |  | 11 | More than 50 % | Cornforth (1995) |
| Suma Wholefoods (wholesaler) | UK | 64 | ?/1977 | 23 | More than 50 % | Jones (2000) |
| 1. Wholegrain Foods: Wholefood, retailing, wholesaling & bakery; | UK | 20 | period studied 1971–1985 | 14 | More than 50 % | Cornforth (1995) |
| **Goal/Cultural regeneration** | | | | | | |
| 1. G (anonymized) baking equipment | Spain (Basque country) | 43 | 1986/1986 | 30 | More than 50 % | Narvaiza et al. (2017) |
| 1. Graficas Verdi, worker recuperated enterprises (ERTs) | Argentina | 30 | 1971/2002 | 5 | More than 50 % | Bryer (2011) |
| 1. M (anonymized) Construction | France (Breton) | 200 | 1986/1986 | 30 | More than 50 % | Narvaiza et al. (2017) |
| 1. New Central Jute Mills | India, Kolkata | 7000 | 1919/1987 | 12 | More than 50 % | Kandathil & Varman (2007) |

**Supplementary Table 5.**

Description of enterprises of the Mondragon Cooperative Cooperation Network

|  | **MONDRAGON** | | | | | | |
| --- | --- | --- | --- | --- | --- | --- | --- |
| Nr. Org. | **Enterprise** | **Country** | **Number of employees** | **Founding year / founding democratic model** | **Tenure of democratic practice (years)** | **Proportion of worker owners** | **Corresponding publications** |
|  | **Degeneration Tendency (Number of enterprises) *n* = 3** | | | | | | |
| 1 | **Fagor Electrodomésticos** group (member of Mondragon CC) (Household appliances) | Spain (Basque country) | 10,543 (2006)  5,673 (2012) | 1956/1956; Nov 2013 bankrupt; | ~56 | less than 50 % | Basterretxea, Heras-Saizarbitoria, & Lertxundi, (2019). |
|  | Fagor Electrodomésticos group (member of Mondragon CC) (Household appliances); Fagor Electrodomésticos parent coop | Spain (Basque country) | 10,543 (2006)  5,673 (2012) | 1956/1956; Nov 2013 bankrupt; | ~56 | less than 50 % | Basterretxea, Cornforth, & Heras-Saizarbitoria, (2022). |
|  | Fagor Electrodomésticos group (founding member of Mondragon CC) (Household appliances); Fagor-Mastercook in Poland (subsidiary)  Fagor-Brandt in France (subsidiary) | Spain (Basque country), international subsidiaries | ~11,000 | Group:1956 / 1956; Mastercook: 1999 / no OD; Brandt: 2002 / no OD | 56 | less than 50 % | Bretos, & Errasti, (2018) |
|  | Fagor Electrodomesticos Group (founding member of MCC); Fagor Electrodomesticos Parent coop household manufacturer; | Spain (Basque country) | Fagor Electrodomenicos group: 10470 (2006), 5500 (2013) (bankruptcy); Fagor Electrodomenicos parent coop: 3850 | 1956 / 1956 | 56 | less than 50 % | Bretos, Errasti, & Marcuello (2019) |
|  | Fagor Electrodomésticos: Polish subsidiary Fagor Mastercook | Poland | 1450 | 1999 / no | 56 | less than 50 % | Bretos, Errasti, & Marcuello (2019) |
|  | Fagor Electrodomésticos: French plants of the subsidiary Fagor Brandt | France | 4400 | 2002 / no | 56 | less than 50 % | Bretos, Errasti, & Marcuello (2019) |
|  | Fagor Electrodomesticos group (member of Mondragon CC) | Spain (Basque country) | 5500 | 1956 / 1956 | 56 | less than 50 % | Errasti, A., Bretos, I., & Etxezarreta, E. (2016) |
|  | Fagor Electrodomesticos: Fagor-Brandt (French subsidiary) | France | 1900 (2012/2013) | Takeover in 2002 | 56 | less than 50 % | Errasti, A., Bretos, & Etxezarreta (2016) |
|  | Fagor Electrodomesticos: Fagor-Mastercook (Polish subsidiary) | Poland | 1750 (2008) | Takeover in the late 1990s | 56 | less than 50 % | Errasti, Bretos, & Etxezarreta (2016) |
|  | Mondragon Cooperative Corporation; Fagor Electrodomesticos (parent coop of the group, member of MCC) | Spain (Basque country) | 5634 (2013) | 1956 / 1956 | 56 | less than 50 % | Errasti, Bretos, & Nunez (2017) |
| 2 | **MAPSA** MCC manufacturing co-op in Pamplona - a maker of aluminum wheels that was converted from a capitalist firm to a cooperative in 1991 and 1992 | Spain (Basque country) | Each coop: 100 - 300 | ? | 2 | ? | Cheney (1997) |
|  | **MAPSA** MCC manufacturing co-op in Pamplona | Spain (Basque country) | ? | ? | 5 | ? | Cheney (2001) |
|  | **MAPSA** MCC manufacturing co-op in Pamplona | Spain (Basque country) | ? | ? | 5 | ? | Cheney (2004) |
| 3 | Mondragon cooperative: **LANA** (agrarian coop; part of EREIN group) | Spain (Basque country) | ~215 | 1960/1960; 1984: exclusion of small farmer members | ~28 | more than 50 % | Taylor (1994) |
|  | **Retention (Number of enterprises) *n* = 1** | | | | | | |
|  | **Longitudinal information (worker owner majority)** | | |  |  |  |  |
| 1 | **Grupo ULMA** (in Ohati), consists of five co-ops that broke away from MCC in early 1992 | Spain (Basque country) | Each coop: 100 - 300 | 1961/1961 | 33 | more than 50 % | Cheney (1997) (see also Bretos et al., 2020) |
|  | **Grupo ULMA** (in Ohati), consists of five co-ops that broke away from MCC in early 1992 | Spain (Basque country) | 5 worker coops with 1.200 employee-owners | 1961/1961 | 36 | more than 50 % | Cheney (2001) |
|  | Grupo ULMA (in Ohati) | Spain (Basque country) |  | 1961/1961 | 36 | more than 50 % | Cheney (2004) |
|  | **Ulma Construction** (member of ULMA coop group?) Construction industry; Ulma Construction subsidiaries (not specified!) | Spain (Basque country) |  | 1961/1961 |  | more than 50 % | Flecha, & Ngai (2014) |
|  | **Degeneration Tendency AND Regeneration (Number of enterprises) *n* = 3** | | | | | | |
| 1 | **Eroski** **supermarkets group** (member of Mondragon CC) | Spain (Basque country) | 52,711 (2008); 33,832 (2014) | 1969 / 1969 | ~46 | less than 50 % | Basterretxea, & Storey (2018) |
|  | Eroski supermarkets group (member of MCC) (see also Basterretxea, & Storey, 2018) retailer Eroski mixed coops | Spain (Basque country) | 33,832 employees (2014) | 1969/1969; From 2009 starting with mixed coops |  | less than 50 % | Flecha, & Ngai (2014) |
|  | Eroski supermarkets (Mondragon CC) | Spain (Basque country) | 43,496 (2011) | 1969/1969 | ~44 | less than 50 % | Storey, Basterretxea, & Salaman (2014) |
| 2 | **Fagor Ederlan group** (8 Coops, 6 subsidiaries; member of MCC); Automotive supplier | Spain (Basque country) | 3559 | Cooperativization: Nov. 2006 - 2008 | Fagor Ederlan Group: 52 | more than 50 % | Bretos, & Errasti (2017) |
|  | Fagor Ederlan Tafalla S. Coop (subsidiary) (Mondragon CC) | Tafalla (Navarre Spain; international) | > 900 | Fagor Ederlan group: 1963 | ~8 | more than 50 % | Bretos, & Errasti (2017) |
|  | Fagor Ederlan Group (Mondragon CC) Automotive supplier foreign subsidiaries: Fagor Ederlan Brasileira (Brazil), Fagor Ederlan Slovensko (Slovakia), and Fagor Ederlan Auto‐Parts Kunshan (China). | Spain (Basque country) | 3600 | 1963/1963 | 49 | more than 50 % | Bretos, & Errasti (2018) |
|  | Fagor Ederlan Group (Mondragon CC) Automotive supplier; International subsidiaries | Spain (Basque country, international subsidiaries) | 3600 | 1963/ 1963 | ~50 | more than 50 % | Bretos, Errasti, & Marcuello (2018) |
|  | Fagor Ederlan Brasileira | Brasil | 440 | 2001 / no OD |  | ? | Bretos, Errasti, & Marcuello (2018) |
|  | Fagor Ederlan Slovakian plant | Slovakia | 170 | 2006 / no OD |  | ? | Bretos, Errasti, & Marcuello (2018) |
|  | Fagor Ederlan Autoparts Kunshan Chinese subsidiary (in the Kunshan Industrial Park) | China | 70 | 2012 / no OD |  | ? | Bretos, Errasti, & Marcuello (2018) |
|  | Fagor Ederlan Parent Coop automotive supplier | Spain (Basque country) | 3600 (2014) | 1963 | ~50 | more than 50 % | Bretos, Errasti, & Marcuello (2019) |
|  | Brazilian subsidiary Fagor Ederlan Brasileira | Brasil | 440 | 2001 / no OD |  | ? | Bretos, Errasti, & Marcuello (2019) |
|  | Slovak subsidiary Fagor Ederlan Slovensko | Slovakia | 170 | 2006 / no OD |  | ? | Bretos, Errasti, & Marcuello (2019) |
|  | Chinese subsidiary Fagor Ederlan Auto-Parts Kunshan (in the Kunshan Industrial Park) | China | 70 | 2012 / no OD |  | ? | Bretos, Errasti, & Marcuello (2019) |
| 3 | Mondragon cooperative **Coprecci** (industrial and home appliances; part of the earlier, meanwhile divided, FAGOR group) | Spain (Basque country) | ~1200 | 1963/1963; 1986: Integration of Copreci into Fagor group | ~25 | more than 50 % | Taylor (1994) |
|  | **Regeneration (Number of enterprises) *n* = 2** | | | | | | |
| 6 | **Maier Group** (member of MCC) Maier Ferroplast mixed coop (subsidiary) | Spain (Basque country) | 2700 (2015);  3200 (2020) | 1975 (founded) /1991 subsidiary / 2012 mixed coop | ~35 | ? | Flecha & Ngai (2014); see also Bretos et al. (2020) |
| 7 | **EB Industrial Cooperative (anonymized; member of MCC)** | Spain (Basque country) | 170 | 1977 | 39 | more than 50 % | Narvaiza et al. (2017) |
|  | **Only general findings used (not specific on organizational level)** | | | | | | |
|  | 11 Mondragon subsidiaries in the **Kunshan Industrial Park**; Owned by their MCC parent coops Wingroup, Batz, Oiarso Medical, Orkli, Orbea, Orona, Fagor Catering, Fagor Metal Forming Machine Tool Co., Fagor Ederlan, Kedi Refrigeration Equipement, Cikautxo | China | 11 subsidiaries: 493 | 7 subsidiaries founded between 2007 and 2008; 4 between 2009 and 2012. | Between 0 to 5 years | ? | Errasti (2015) |
|  | **11 Mondragon (MCC) parent cooperatives**: Corporation Finance; industrial sector; distributional sector (**coop names not specified!**) | Spain (Basque country | ? | majority > 5 | ? | ? | Heras-Saizarbitoria (2014) |
|  | **Mondragon Corporation: 70 cooperatives** | Spain (Basque country | ? | 1956 | 60 | ? | Heras-Saizarbitoria & Basterretxea (2016) |
|  | **Mondragon Corporation** | Spain (Basque country | 75000 | 1955/1955 | 63 |  | Hoffman & Shipper (2018) |
|  | **Mondragon Corporation** | Spain (Basque country | 85322 (2011) | 1956/1956 | ~56 | more and less than 50 % depends on the single coop group | Blawat (2014) |
